# Screening and Evaluation In Vitro of *Bacillus*-Based Probiotics for Feed Additives

**DOI:** 10.3390/microorganisms14040834

**Published:** 2026-04-07

**Authors:** Yujun Mao, Xiaofang Lou, Jianmei Che, Xiaoyun Huang, Yanping Chen, Jianglin Lan, Meichun Chen, Xin Liu, Qinlou Huang, Xiusheng Huang, Jieping Wang

**Affiliations:** 1 College of Life Sciences, Fujian Agriculture and Forestry University, Fuzhou 350002, China; yujunmao2026@163.com; 2 Fujian Engineering and Technology Research Center for Recycling Agriculture in Hilly Areas, Institute of Resources, Environment and Soil Fertilizer, Fujian Academy of Agricultural Sciences, Fuzhou 350003, China; 18208447534@163.com (X.L.); chejm2002@163.com (J.C.); huangxy364@163.com (X.H.); chenyanping@faas.cn (Y.C.); lanfz2008@163.com (J.L.); cmczjw@163.com (M.C.); fzliuxin@yeah.net (X.L.); hql202@126.com (Q.H.); 3 College of Animal Science, Fujian Agriculture and Forestry University, Fuzhou 350002, China

**Keywords:** *Bacillus velezensis*, *Bacillus*-based probiotics, probiotic properties, safety assessment, whole-genome analysis, feed additives

## Abstract

In the post-antibiotic era, the *Bacillus*-based direct-fed beneficial microorganisms are emerging as a cornerstone for sustainable animal farming. This study aimed to screen and evaluate *Bacillus* strains with probiotic potential for use as feed additives. A total of 394 *Bacillus* strains were initially screened based on their extracellular enzyme production (cellulase, protease, and amylase) and antibacterial activities against *Escherichia coli*, *Staphylococcus aureus*, and *Salmonella enterica*. Two strains, *Bacillus velezensis* FJAT-10508 and FJAT-13563, were selected and subsequently subjected to in vitro probiotic characterization, safety assessment, and whole-genome analysis. The results demonstrated that both strains exhibited α-hemolysis, acceptable antibiotic susceptibility profiles, absence of invasion and cytotoxicity effect on the Caco-2 cells, and no mobile virulence or antibiotic resistance genes, indicating their safety as probiotic candidates. High endospore-forming efficiencies (72.4–90.8%), strong auto-aggregation (74–85%) and co-aggregation abilities (52–82%) were observed. In addition, both strains showed considerable tolerance to simulated gastrointestinal conditions, with vegetative cell and endospore survival rates of 28.33–38.33% and 85–89.67% at pH 2.0, and 38.33–43.33% and 90.33–96.33% in 0.3% bile salts, respectively. Overall, *B. velezensis* FJAT-10508 and FJAT-13563 demonstrated robust in vitro probiotic properties, supporting their potential application as reliable *Bacillus*-based feed additives.

## 1. Introduction

In the livestock and poultry industry, antibiotics are not only extensively used to treat bacterial infections but also employed as non-therapeutic growth promoters to enhance the production efficiency of animals [1,2]. However, long-term and excessive use of antibiotics has resulted in significant issues, including antibiotic residue contamination of livestock products and the emergence of antimicrobial-resistant bacteria (ARB) [3]. Furthermore, the development and horizontal transfer of antibiotic-resistant genes (ARGs) among bacteria, the disruption of normal microbiota, and the corresponding decline in beneficial intestinal bacteria are all associated with the overuse of antibiotics [2,4]. These concerns have intensified global efforts to reduce antibiotic dependence in animal production systems. Therefore, identifying antibiotic alternatives that do not compromise animal production performance or escalate industry costs has become a critical challenge for sustainable animal farming. Currently, the most widely utilized antibiotic alternatives include enzymes, acidifiers, antimicrobial peptides, phages, probiotics, and plant extracts. Among these alternatives, probiotics administered in the form of live bacteria can directly colonize the intestine and rapidly exert beneficial effects through mechanisms such as ecological niche occupation, organic acid production, and antimicrobial substance synthesis [5,6].

The Food and Agriculture Organization of the United Nations/World Health Organization (FAO/WHO) defines probiotics as follows: “Probiotics are a category of live microorganisms. When an adequate number of these live bacteria reach the host’s intestines and successfully establish themselves, they can modify the balance of the host’s intestinal microbiota, thereby exerting a beneficial health effect on the host.” [7]. Therefore, direct-fed beneficial microorganisms are emerging as a cornerstone for sustainable animal farming in the post-antibiotic era [8]. Currently, the commonly used probiotics in feed and animal husbandry mainly originate from lactic acid bacteria, *Bifidobacteria*, and the *Bacillus*-like bacteria [9]. However, beneficial microorganisms such as lactic acid bacteria and *Bifidobacteria* generally exhibit limited tolerance to the high temperatures encountered during feed pelletization, resulting in reduced shelf-life and compromised efficacy in conventional animal production systems [10]. In contrast, *Bacillus*-like bacteria have attracted increasing attention due to their superior stability and functional versatility [11,12]. These bacteria can enhance animal growth performance, optimize feed utilization efficiency, exhibit antioxidant properties, and help maintain intestinal microbial balance [13,14]. Importantly, their endospore-forming capability confers resistance to harsh processing, storage conditions, and gastrointestinal transit [15,16]. For instance, Ramulken et al. [17] demonstrated that *Bacillus* endospores could retain approximately 90% viability during probiotic harvesting, with a potential shelf-life of up to five years. These attributes make *Bacillus*-like bacteria particularly suitable for modern feed industry applications. Among them, the species *Bacillus velezensis*, which is widely used a plant growth-promoting bacterium and biocontrol agent of plant pathogens, is now increasingly utilized as a direct-fed probiotic in animal feed [18,19].

Numerous studies have indicated that the *Bacillus*–like bacteria can secrete extracellular digestive enzymes, which can significantly increase the daily weight gain and growth rate of animals by improving the digestibility of feed [20,21,22]. Currently, the commonly used digestive enzymes on the market include cellulase, amylase, and protease. Cellulase breaks down the β-1, 4 glycosidic bonds in cellulose within the cell walls of grains, destroying their structure and releasing absorbable nutrients such as glucose, thereby enhancing the energy value of feed and the absorption and utilization rate of cellulose by animals [23]. The addition of protease can further enhance the utilization rate of protein, meeting the rapid growth demands of animals for amino acids [24]. Alpha-amylase can randomly cleave the α-1, 4 bonds in starch, rapidly reducing viscosity and generating maltodextrins, thereby increasing the metabolic energy of animals [25]. Thus, supplementing diets with selected enzymes can effectively counter the poor degradability of highly structured feed components, especially in monogastric animals [26]. By depolymerising non-starch polysaccharides, exogenous enzymes can lower digest viscosity and release low-molecular-weight carbohydrates, thereby improving nutrient utilization [27,28]. *Bacillus* spp. contribute to this process through the secretion of extracellular enzymes (such as cellulases, amylases, and proteases), which enhance the accessibility of otherwise refractory nutrients [20] and elevate the energy value of the diet, resulting in greater feed efficiency [29,30]. For example, *Bacillus subtilis* KT004404, capable of producing multiple enzymes, significantly improved feed conversion efficiency and weight gain in broilers [20].

Furthermore, *Bacillus*-like bacteria can modulate the intestinal environment, suppressing pathogenic microorganisms while promoting beneficial microbial populations [11,31,32,33]. These effects are mediated by the production of bioactive compounds, including bacteriocins, organic acids, and secondary metabolites, such as lipopeptides, which inhibit pathogens such as *Escherichia coli* (*E. coli*), *Staphylococcus aureus* (*St. aureus*), and *Salmonella enterica* (*Sa. enterica*) [34,35]. For example, oral administration of *B. subtilis* GX15 significantly alleviated clinical symptoms and reduced intestinal and hepatic tissue damage in *Salmonella*-infected mice [36].

In this study, the *Bacillus*-based probiotic candidates were screened from a repository of 394 isolates, according to their extracellular-enzyme-producing abilities and antibacterial activities against *E. coli*, *St. aureus* and *Sa. enterica*. Subsequently, their probiotic credentials and safety indices, including aggregation, sporulation efficiency, acid/bile-salt tolerance, antibiotic susceptibility, prediction of putative virulence factors and ARGs, and haemolytic potential, were systematically evaluated. Our work would thereby provide probiotic candidates for formulating *Bacillus*-based feed additives.

## 2. Materials and Methods

### 2.1. Strains and Growth Conditions

The 394 *Bacillus* strains (listed in Table S1) and the pathogen strains (*E. coli* ATCC 25922, *St. aureus* ATCC 25923, and *Sa. enterica* ATCC 14028) used in this study were all preserved in the Fujian Bacilli Resource Collection Center (FBRCC), Institute of Resources, Environment, and Soil Fertilization, Fujian Academy of Agricultural Sciences. The taxonomic identification of each *Bacillus* strain was conducted by FBRCC through 16S rRNA and *gyrB* sequence analyses. Unless otherwise stated, all reagents used in this study were purchased from China National Biotec Group Co., Ltd., Beijing, China.

The *Bacillus* and pathogenic strains were all cultured using Luria–Bertani (LB) media (10 g tryptone, 5 g yeast extract, 10 g NaCl per 1 L distilled water, pH 7.0). Briefly, each strain was initially streaked onto a LB agar plate and incubated at 37 °C for 24 h to obtain single colonies. A single colony was then inoculated into LB broth and cultured overnight (14–16 h) at 37 °C with shaking at 180 rpm.

To harvest endospores, a single colony of each *Bacillus* strain was inoculated into Schaeffer’s sporulation medium (SSM) [8 g nutrient broth, 0.12 g MgSO_4_, 1 g KCl, 0.5 mM NaOH, 1 mM Ca(NO_3_)_2_, 0.01 M MnCl_2_, and 1 M FeSO_4_ per 1 L distilled water, pH 7.0] [37] and cultured at 37 °C with shaking at 180 rpm for 48 h.

### 2.2. Screening of Extracellular Digestion Enzyme-Producing Bacillus Strains

All the 394 *Bacillus* strains were initially assessed their extracellular cellulase-, amylase-, and protease-producing capabilities by using the transparent ring method. Firstly, each *Bacillus* strain was inoculated into 5 mL LB broth and grown overnight at 37 °C with shaking at 180 rpm. Each obtained culture was adjusted to 1.0 × 10^8^ CFU·mL^−1^ (OD_600nm_ = 0.5). Subsequently, 2 μL cultures of each *Bacillus* strain were dropped onto the surface of the cellulose agar (10 g sodium carboxymethyl cellulose (CMC-Na), 5 g yeast extract, 5 g peptone, 5 g NaCl, 15 g agar per 1 L distilled water, pH 7.0), starch agar (3 g beef extract, 10 g peptone, 20 g soluble starch, 10 g agar per 1 L distilled water, pH 7.0), and skimmed milk agar (3 g beef extract, 10 g peptone, 50 g skimmed milk powder, and 10 g agar per 1 L distilled water, pH 7.0) plates (90 mm) with three replicates per strain alongside a blank control, respectively. These plates were incubated statically at 37 °C for two days to observe whether any hydrolysis rings had formed (iodine solution was added to visualize starch hydrolysis rings). Finally, the diameters of colonies (d) and hydrolysis rings (D) were measured with a digital caliper. The D/d ratio value was used to preliminarily estimate extracellular cellulose-, amylase-, and protease-producing activities of each *Bacillus* strain.

### 2.3. Screening of the Bacillus Strains with Antibacterial Activities Against Three Pathogens

The double-layer agar method was employed to preliminarily assess the antibacterial activity of each *Bacillus* strain against three common pathogens: *E. coli*, *St. aureus*, and *Sa. enterica* found in livestock and poultry. Firstly, each *Bacillus* strain and pathogenic strain was separately inoculated into 5 mL LB broth and grown overnight at 37 °C with shaking at 180 rpm. Semi-solid LB agar media containing 200 μL of pathogenic cultures (1.0 × 10^8^ CFU·mL^−1^) were poured onto the Petri plates (90 mm) coated with LB solid media. After solidification, 2 μL cultures (1.0 × 10^8^ CFU·mL^−1^) of each *Bacillus* strain were dropped onto the surface of the two-layer plates with three replicates per strain, and then co-cultivated at 37 °C for one or two days. The clear zone formed around the *Bacillus* colonies indicated the growth inhibition of the tested pathogen, and then the diameters (mm) of the inhibition zones were measured using a digital calliper to evaluate the inhibitory capacity of each *Bacillus* strain against different pathogenic bacteria.

Comprehensively considering the analysis results of extracellular-enzyme producing abilities and antibacterial activities against *E. coli*, *St. aureus*, and *Sa. enterica*, the strains FJAT-10508 and FJAT-13563 were ultimately selected for further studies.

### 2.4. Genome Sequencing and Analyses of FJAT-10508 and FJAT-13563

Genome sequencing, assembly, and annotation were all performed by Shanghai Majorbio Biopharmaceutical Technology Co., Ltd. (Shanghai, China) and genomic analyses were performed through the online tools of Majorbio Cloud Platform (https://www.majorbio.com/tools, accessed on 26 December 2025) [38]. Briefly, genomic DNA from FJAT-10508 and FJAT-13563 was separately extracted from overnight cultures grown at 37 °C in LB broth using the Promega Wizard Genomic DNA Purification Kit (Madison, WI, USA). The concentration, quality, and integrity of genomic DNA were determined using a Qubit Fluorometer and a NanoDrop spectrophotometer (Thermo Fisher Scientific, Waltham, MA, USA), respectively. For genome sequencing, PacBio RS II (Pacific Biosciences, Menlo Park, CA, USA) and Illumina NovaSeq X Plus (Illumina, San Diego, CA, USA) platforms were used. Hybrid reads were assembled de novo using Unicycler (SPAdes v 4.0.0). The gene prediction and functional annotation were performed with Glimmer, followed by BLAST searches against the NCBI Non-Redundant Protein Database (NR; https://ftp.ncbi.nlm.nih.gov/blast/db, accessed on 26 December 2025), Swiss-Prot (https://www.expasy.org/resources/uniprotkb-swiss-prot, 26 December 2025), Clusters of Orthologous Groups of proteins (COG; https://www.ncbi.nlm.nih.gov/research/cog-project/, accessed on 26 December 2025), Gene Ontology (GO; https://www.geneontology.org, accessed on 26 December 2025), and the Kyoto Encyclopedia of Genes and Genomes (KEGG; https://www.genome.jp/kegg/, accessed on 26 December 2025).

Putative virulence genes and AGRs were predicted by using the Virulence Factor Database (VFDB) [39] and the Comprehensive Antibiotic Resistance Database (CARD) [40], respectively. A pangenome analysis of 435 *B. velezensis* genomes (including all NCBI complete genomes and FJAT-10508 and FJAT-13563) was performed using PGAP2 (https://github.com/bucongfan/PGAP2, accessed on 26 December 2025) [41], and the putative virulence genes of FJAT-10508 and FJAT-13563 were mapped to pangenome categories, respectively. Mobilome (plasmids, prophages, insertion sequences, inverted repeat elements, and compositional outlier regions) annotation of the FJAT-10508 and FJAT-13563 genomes was performed by using the Mobilome Annotation Pipeline developed by EBI-Metagenomics (https://github.com/EBI-Metagenomics/mobilome-annotation-pipeline, accessed on 26 December 2025). Biosynthetic gene clusters (BGCs) potentially involved in antimicrobial metabolite production were searched using antiSMASH 7.0 [42]. The average nucleotide identity (ANI) values of strains FJAT-10508 and FJAT-13563 with phylogenetically related strains were calculated using the Ortho ANIu algorithm [43], with an ANI cut-off of 96% for species delineation [44].

### 2.5. Activity Determination of FJAT-10508 and FJAT-13563 Antibacterial Compounds Enriched by Acid Precipitation

Firstly, the cell-free supernatants of FJAT-10508 and FJAT-13563 were harvested from the cultures in LB broth at 25 °C with shaking at 180 rpm for 48 h by centrifugation (4000× *g*, 10 min, 4 °C) and adjusted to pH 2.0 with 3 M HCl to precipitate antibacterial compounds, respectively. After overnight storage at 4 °C, the precipitates were collected by centrifugation (10,000× *g*, 10 min, 4 °C), re-dissolved in phosphate-buffered saline solution (PBS, pH 6.8), lyophilized, and stored as a dry powder at –20 °C until required. Immediately prior to bioassays, the lyophilized powder was dissolved in sterile ddH_2_O to prepare a solution of the antibacterial compound at a final concentration of 30 mg·mL^−1^ for antibacterial activity testing.

Antibacterial activities of the compounds enriched by acid precipitation from FJAT-10508 and FJAT-13563 against *E. coli*, *St. aureus* and *Sa. enterica* were evaluated using the agar-well diffusion method [45]. Briefly, 100 μL cultures (1 × 10^8^ CFU·mL^−1^) of *E. coli*, *St. aureus* and *Sa. enterica* at the mid-log phase were evenly spread onto the LB agar plates, respectively. After surface drying, 9 mm-diameter wells were aseptically punched into the agar, and then 100 µL of an antibacterial compound solution (30 mg·mL^−1^), cell-free supernatant, bacterial cell suspension, or whole culture broth was separately loaded into each 9 mm well. Fresh LB broth and streptomycin sulphate (200 µg·mL^−1^) were used as the negative and positive controls, respectively. The plates were incubated at 37 °C for 48 h, and the diameters of inhibition zones were measured using a digital caliper.

### 2.6. Determination of Aggregation Activities of FJAT-10508 and FJAT-13563

The autoaggregation and co-aggregation capabilities, phenotypic traits for the screening of potential probiotic strains related to the adherence capability to intestinal epithelial cells, were determined according to the procedure of Reuben et al. [46] with some minor modifications. Bacterial suspensions used in these assays were harvested at the stationary phase (after 24 h of incubation) and adjusted to approximately 1 × 10^8^ CFU·mL^−1^. In the autoaggregation assays, 4 mL of each vortexed *Bacillus* culture suspension (1 × 10^8^ CFU·mL^−1^) was statically incubated at 37 °C, the absorbance was separately measured by a spectrophotometer (BioTek Instruments, Inc., Winooski, VT, USA) at 0 h (initial optical density, OD_i_), and 3, 5 and 24 h (optical density at certain time, OD_t_). The autoaggregation percentage (%) was calculated as [1 − (OD_t_/OD_i_)] × 100. As for the co-aggregation assays, 2 mL of each *Bacillus* and pathogen culture suspensions were mixed, vortexed, and statically incubated at 37 °C for 3, 5 and 24 h, with 4 mL of each *Bacillus* or pathogen culture suspension alone as control treatment. At a certain time, the absorbance of each mixed suspension was measured at 600 nm (OD_mix_) and compared with those of the control treatments containing *Bacillus* (OD*_Bacillus_*) and pathogen (OD_pathogen_) alone. The co-aggregation percentage was (%) was calculated as [1 − OD_mix_/(OD*_Bacillus_* + OD_pathogen_)/2] × 100.

### 2.7. Invasion and Cytotoxicity Assays of FJAT-10508 and FJAT-13563 to Caco-2 Cells

Invasion and cytotoxicity effect to Caco-2 cells of FJAT-10508 and FJAT-13563 were evaluated according to the previously described methods [47,48] with some modifications. CACO-2 cells were cultured in RPMI-1640 medium supplemented with 10% fetal bovine serum (FBS; Gibco, Grand Island, NY, USA), and maintained at 37 °C in a humidified atmosphere containing 5% CO_2_. Upon reaching 80–90% confluence, the CACO-2 cells were detached using 0.25% trypsin-EDTA (Gibco).

For invasion assays, FJAT-10508 and FJAT-13563 were cultured in LB broth at 37 °C with shaking at 180 r·min^−1^ for 24 h. Subsequently, 100 μL of the CACO-2 cell suspension (5 × 10^4^ cell·mL^−1^) was mixed with 100 μL of FJAT-10508 or FJAT-13563 culture suspension (2.5 × 10^5^ CFU·mL^−1^), with a ratio of approximately 50:1 (bacteria: mammalian cells) for the multiplicity of infection (MOI), and loaded into 48-well plates. The mixture was incubated at 37 °C in a 5% CO_2_ atmosphere for 1 h. After incubation, non-invasive bacteria were removed by washing with PBS, and the Caco-2 cells were treated with 1 mL of gentamicin (100 mg·mL^−1^ in PBS) per well and incubated at 37 °C for another 1 h. The cells were then washed twice with PBS and lysed in 1 mL of sterile distilled water at 37 °C for 1 h to release the invading bacteria. After serial dilution, the samples were spread onto the LB plates for bacterial cell counting. Invasion ability was expressed as the percentage of bacteria recovered after gentamicin treatment relative to the total number of initial cells.

For cytotoxicity assays, CACO-2 cells were seeded into 96-well plates at a density of 5 × 10^4^ cell·mL^−1^ (200 μL per well) and incubated for 24 h. FJAT-10508 and FJAT-13563 were cultured in LB broth at 37 °C with shaking at 180 rpm for 18 h. Bacterial cultures were centrifuged at 3000× *g* for 10 min at 4 °C, and then filtered through a 0.22 μm filter membrane to obtain cell-free culture supernatants. For the experimental group, 100 μL of the cell-free culture supernatant was added to each well. For the negative control group, PBS was added instead of the bacterial supernatant. For the positive control group, 1% Triton X-100 was added to induce complete cell lysis. Blank controls contained only RPMI-1640 medium without cells. After co-incubation for 18 h at 37 °C, the supernatant was removed, and 10 μL of CCK-8 solution (1 mg·mL^−1^ in PBS) was added to each well, followed by incubation for an additional 1 h at 37 °C. The absorbance was measured at 450 nm using a microplate reader (BioTek Instruments, Inc., Winooski, VT, USA). Cell viability was calculated as: Cell viability (%) = (OD_experimental_ − OD_blank_)/(OD_negative_ − OD_blank_) × 100.

### 2.8. Detection of Endospore-Forming Efficiencies of FJAT-10508 and FJAT-13563

The strains FJAT-10508 and FJAT-13563 were separately inoculated into 5 mL of SSM broth and cultured at 37 °C with shaking at 180 rpm. The cultures were harvested at 24, 48, and 72 h, respectively. Each harvested culture was divided into two portions; one was heated at 80 °C for 20 min to kill the vegetative cells that did not form spores, and the other was not. Finally, the CFU value in each sample was determined using the plate colony-counting method. The endospore-forming efficiency (%) was calculated as CFU_heated_/CFU_non-heated_ × 100.

### 2.9. Simulated Gastrointestinal Tract Tolerance of FJAT-10508 and FJAT-13563

Tolerance of vegetative cells and endospores of FJAT-10508 and FJAT-13563 to simulated gastrointestinal conditions and bile salt was determined as described by Unban et al. [38] with some modifications. Firstly, the strains FJAT-10508 and FJAT-13563 were inoculated into 10 mL LB broth to prepare vegetative cells in the mid log-phase (37 °C, 180 rpm, 12–16 h), and SSM broth to harvest the mixture of vegetative cells and endospores (37 °C, 180 rpm, 48 h) and pure endospores by heating at 80 °C for 20 min, respectively. For artificial gastric and intestinal juices tolerance testing of vegetative cells, the cells were harvested by centrifugation (8000× *g*, 10 min, 4 °C), washed once, and resuspended in 5 mL of fresh LB acidified to pH 2.0 and supplemented with pepsin (1 mg·mL^−1^, Solarbio Co., Ltd., Beijing, China; 1:3000; Cat. No. P8390), and fresh LB and supplemented with 1 mg·mL^−1^ pancreatin (Solarbio Co., Ltd., Beijing, China; 1:250; Cat. No. T8150) and 0.3% (*w*/*v*) bile salts, respectively. For tolerance testing of pure endospores and mixture, each sample was centrifugated (8000× *g*, 10 min, 4 °C), washed once, and resuspended in 5 mL of saline solution (NaCl, 0.9%) adjusted to pH 2.0 and supplemented with 1 mg/mL of pepsin, or an isotonic buffer [Bott and Wilson salts: 1.24% K_2_HPO_4_, 0.76% H_2_PO_4_, 0.1% trisodium citrate, 0.6% (NH_4_)_2_SO_4_, pH 6.7] with 1 mg/mL of pancreatin and 0.3% bile salts. Each mixed sample was incubated at 37 °C with shaking at 120 rpm, and samples were collected after 30, 60 and 90 min for artificial gastric fluid tolerance and after 60 and 180 min for artificial intestinal fluid tolerance. The working concentration of each vegetative cell, mixture, and pure endospore sample was set as 1 × 10^7^ CFU·mL^−1^ (CFU_initial_). At each time point, the viable CFU value (CFU_viable_) of each sample was determined using the plate colony-counting method. The survival rate (%) was calculated as logCFU_viable_/logCFU_initial_ × 100.

### 2.10. Antibiotic Sensitivity Test of FJAT-10508 and FJAT-13563

The strains FJAT-10508 and FJAT-13563 were evaluated their sensitivity to ampicillin (10 μg per disc), cefaclor (30 μg per disc), chloramphenicol (30 μg per disc), enrofloxacin (10 μg per disc), erythromycin (15 μg per disc), gentamicin (10 μg per disc), kanamycin (30 μg per disc), neomycin (30 μg per disc), polymyxin B (300 IU per disc), streptomycin (10 μg per disc), sulfamethoxazole (300 μg per disc), and tetracycline (30 μg per disc) (Hang Zhou Microbial Reagent Co., Ltd., Hangzhou, China). Briefly, 100 µL of FJAT-10508 and FJAT-13563 culture suspensions were poured onto LB agar plates, and the aforementioned commercial antibiotic disks were added to each plate (4 disks per plate). These plates were incubated in a dark incubator at 37 °C for 24–48 h. The zone of inhibition around each drug susceptibility disk was measured using a digital caliper, and the antibiotic susceptibility was interpreted according to CLSI M100-2025, Performance Standards for Antimicrobial Susceptibility Testing, 35th ed. CLSI, Wayne, PA, USA, 2025.

If the result of the susceptibility disc test was resistant (R) for one antibiotic, its minimum inhibitory concentration (MIC, the lowest concentration that inhibited ≥90% of visible growth compared with the drug-free control) was determined by the broth microdilution method following the CLSI guidelines. Briefly, the log–growth-phase cultures of FJAT-10508 and FJAT-13563 grown in Mueller–Hinton broth (MHB; Solarbio, Beijing, China) were adjusted to a concentration of approximately 5 × 10^5^ CFU·mL^−1^ to prepare the strain working solutions using fresh MHB. Each tested antibiotic underwent a two-fold serial dilution in MHB to yield a final concentration ranging from 0.25 to 16 µg·mL^−1^. Aliquots (100 µL) of each dilution were dispensed into 96-well polystyrene microtitre plates, followed by the addition of an equal volume of the strain working solution to achieve a final inoculum of approximately 2.5 × 10^5^ CFU·mL^−1^ per well (ODs). Negative control (the strain working solution and MHB were mixed at equal volume without any antibiotic, ODn) and blank controls (MHB broth alone, ODb) were dispensed in the plates. After sealing, the plates were incubated at 30 °C for 24 h, and the absorbance at 600 nm (OD_600nm_) was measured using a microplate reader (BioTek Instruments, Inc., Winooski, VT, USA). All assays were performed in triplicate. The inhibition rate (IA) was calculated as follows: IA (%) = [(ODn − ODb) − (ODs − ODb)]/(ODn − ODb) × 100.

### 2.11. Hemolysis Activity Assay of FJAT-10508 and FJAT-13563

One microliter of a mid-log phase culture (1 × 10^8^ CFU·mL^−1^) of FJAT-10508 and FJAT-13563 was spotted onto Columbia blood agar (5% sheep blood per plate; Shanghai Shenqi Bio-technology Co., Ltd., Shanghai, China). *St. aureus* FJAT-2450 and fresh LB broth served as the positive and negative controls, respectively. Plates were incubated at 30 °C for 48 h to examine the presence of hemolysis zones surrounding the colonies. Hemolytic patterns were recorded as α-hemolysis (greenish zone), β-hemolysis (clear zone) or γ-hemolysis (no zone).

### 2.12. Statistical Analysis

All experiments were repeated three times. All data were presented as the mean ± standard error (SEM). Prior to parametric analysis, the normality of data distribution was assessed using the Shapiro–Wilk test. For data that followed a normal distribution, one-way ANOVA was performed using SPSS v21.05 (Hangzhou Ruifeng Information Technology Co., Ltd., Hangzhou, China) followed by Duncan’s multiple range test for post-hoc comparisons. For data that did not meet the normality assumption, the Kruskal–Wallis test was used instead, followed by Dunn’s test for multiple comparisons. Significant differences were determined at *p* ≤ 0.05.

## 3. Results

### 3.1. Extracellular-Enzyme-Producing Abilities of the 394 Bacillus Strains

**Figure 1:**
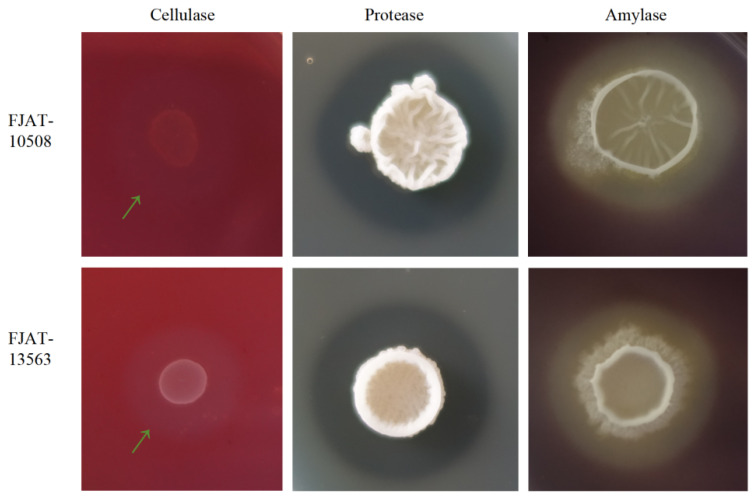
Extracellular enzyme-producing abilities of FJAT-10508 and FJAT-13563. Notes: The extracellular cellulose-, protease-, and amylase-producing capabilities were initially assessed by using the transparent ring method on the cellulose, skimmed milk, and starch agar plates (90 mm), respectively. The hydrolysis ring edge of cellulose was indicated by a green arrow. The diameters of colonies (d) and hydrolysis rings (D) were measured with a digital caliper. The D/d ratio value was used to preliminarily estimate the producing ability of each extracellular enzyme.

To screen probiotic candidates for formulating *Bacillus*-based feed additives, the extracellular-enzyme-producing abilities of the 394 *Bacillus* strains were evaluated using the transparent ring method first. The results showed that 203, 305, and 158 *Bacillus* strains exhibited extracellular cellulase, protease, and amylase-producing abilities, with the average D/d ratio value ranges of 1.33–6.9, 1.09–3.5, and 1.12–2.45, respectively (Table S1). Moreover, 128 out of 394 *Bacillus* strains could produce all three extracellular digestion enzymes with varying degrees. Among these 128 strains, the comprehensive abilities of FJAT-10508 and FJAT-13563 were relatively remarkable, with the average D/d ratio values of 1.92 and 2.13 for cellulase, 1.5 and 1.91 for protease, and 1.75 and 1.56 for amylase, respectively (Figure 1).

### 3.2. Antibacterial Activities of the 394 Bacillus Strains Against E. coli, St. aureus and Sa. enterica

The antibacterial activities of the 394 *Bacillus* strains against three common pathogens in livestock and poultry were further evaluated by the double-layer agar method. The results indicated that 20, 7, and 5 *Bacillus* strains displayed antibacterial activities against *E. coli*, *St. aureus* and *Sa. enterica* under the conditions of this study, with the average diameters of inhibition zone ranging from 11.35 to 19.84 mm, 12.32 to 16.76 mm and 11.32 to 17.1 mm, respectively (Table S1). Fortunately, FJAT-10508 and FJAT-13563 were the only two strains that simultaneously exhibited inhibitory activity against three pathogens, with average inhibition zone diameters of 16.02 and 14.36 mm for *E. coli*, 16.76 and 13.96 mm for *St. aureus*, and 12.6 and 11.32 mm for *Sa. enterica*, respectively. Consequently, the strains FJAT-10508 and FJAT-13563 were selected to further evaluate probiotic properties in vitro, based on the comprehensive consideration of extracellular-enzyme producing abilities and antibacterial activities against *E. coli*, *St. aureus* and *Sa. enterica*.

### 3.3. Genomic Evaluation of the Strains FJAT-10508 and FJAT-13563

According to the guidelines of the European Food Safety Authority (EFSA), whole-genome evaluation for the strains FJAT-10508 and FJAT-13563 was performed, which mainly focused on taxonomic identification of the strains, prediction, and potential mobility assessment of putative virulence genes and AGRs, and identification of BGCs for antimicrobial metabolites. Generally, the results showed that the FJAT-10508 and FJAT-13563 genomes consisted of one circular chromosome of 3,929,792 bp and 4,080,379 bp, with the average G + C content of 46.5% and 46.35%, respectively (Figure S1). No plasmid was discovered in either genome. A total of 3529 and 3576 protein-coding genes were predicted in the FJAT-10508 and FJAT-13563 genomes, respectively. Genome sequences of the strains FJAT-10508 and FJAT-13563 were deposited in GenBank under the accession numbers PRJNA1397014 and PRJNA1397016, respectively.

**Figure 2:**
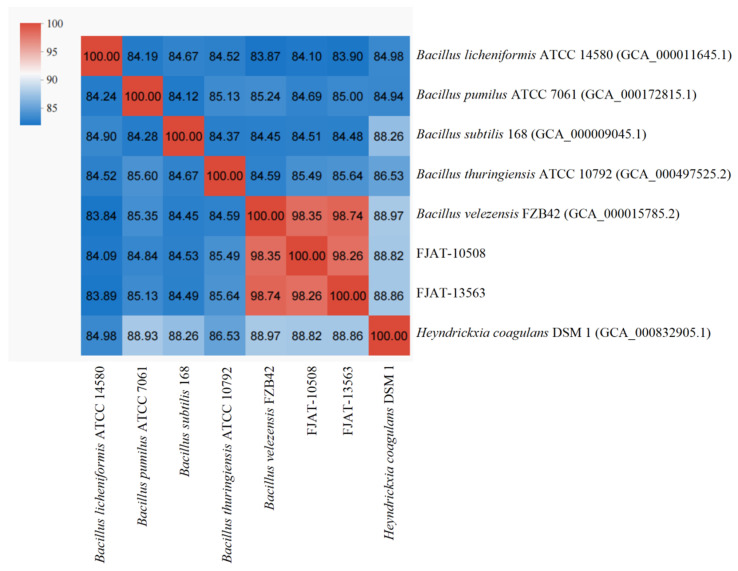
Heatmap of the strains FJAT-10508 and FJAT-13563 with phylogenetically related *Bacillus* strains based on the ANI values. Notes: The heatmap analysis was performed using the online tool on the Majorbio Cloud Platform (https://www.majorbio.com/tools, accessed on 26 December 2025) [38]. The phylogenetically-related *Bacillus* strains and their genomes included *Bacilluus licheniformis* ATCC 14580 (GCA_000011645.1), *B. pumilus* ATCC 7061 (GCA_000172815.1), *B. subtilis* 168 (GCA 000009045.1), *B. thuringiensis* ATCC 10792 (GCA_000497525.2), *B. velezensis* FZB42 (GCA 000015785.2), and *Heyndrickxia coagulans* DSM 1 (GCA_000832905.1).

Firstly, the strains FJAT-10508 and FJAT-13563 were further identified by whole-genome ANI analysis, although the taxonomic status had been validated previously by FBRCC using 16S rDNA and *gyrB* gene sequence analyses. The results showed that the ANI values of the strains FJAT-10508 and FJAT-13563 with the strain *B. velezensis* FZB42 were higher than 98.35%, exceeding the ANI cut-off value of 96% for species delineation, while those with the strains *B. licheniformis* ATCC 14580, *B. pumilus* ATCC 7061, *B. subtilis* 168, *B. thuringiensis* ATCC 10792 and *Heyndrickxia coagulans* (formerly *Bacillus coagulans*) DSM 1 were all lower than 88.9% (Figure 2). Therefore, both FJAT-10508 and FJAT-13563 were validated again as members of the Species *B. velezensis*.

Subsequently, the FJAT-10508 and FJAT-13563 genomes underwent searches against the databases VFDB and CARD to predict the putative virulence factor and ARG-related genes, respectively. A total of 226 and 231 putative virulence genes were predicted in the FJAT-10508 and FJAT-13563 genomes, respectively, which are mainly involved in adherence, effector delivery systems, exoenzymes, exotoxins, immune modulation, invasion, and stress survival (Table S2). The FJAT-10508 and FJAT-13563 genomes were predicted to hold 160 and 148 putative ARGs, which were related to 38 categories of antibiotics, such as aminoglycosides, cephalosporins, fluoroquinolones, glycopeptides, macrolides, phenicols, rifamycins, and tetracyclines (Table S3).

To evaluate the potential mobility of the putative virulence factors and AGRs, the FJAT-10508 and FJAT-13563 genomes were further annotated for the mobilome (plasmids, prophages, insertion sequences, inverted repeat elements, and compositional outlier regions) using the Mobilome Annotation Pipeline. The results showed that most of the predicted virulence genes and AGRs (343 out of 386 for FJAT-10508, 88.86%; 351 out of 379 for FJAT-13563, 92.61%) had no mobile genetic element within their flanking 100 bp regions (Table S4), implying that these putative virulence factors and ARGs were predominantly intrinsic to both genomes, but not mobile.

To evaluate whether the putative virulence genes are typical, low-risk housekeeping, or high-risk features, 435 *B. velezensis* genomes (including the FJAT-10508 and FJAT-13563 genomes) were used to perform a pangenome analysis using PGAP2, and the putative virulence genes of FJAT-10508 and FJAT-13563 were mapped to the pangenome categories, respectively. The results revealed that the Species *B. velezensis* exhibited an open pangenome with 27,791 total genes, and 84.04% cloud genes, 5.87% core genes, 5.18% shell genes, 3.43% soft core genes, and only 1.48% strict core genes (Table S5). The putative virulence genes annotated by VFDB of FJAT-10508 and FJAT-13563 exhibited the pangenome categories of 56.34% and 57.95% cloud genes, 16.9% and 17.33% core genes, 10.42% and 8.52% shell genes, 6.2% and 5.97% soft core genes, and 10.14% and 10.23% strict core genes, respectively (Figure S2).

### 3.4. Preliminary Evaluation of the Antibacterial Compounds from FJAT-10508 and FJAT-13563

**Figure 3:**
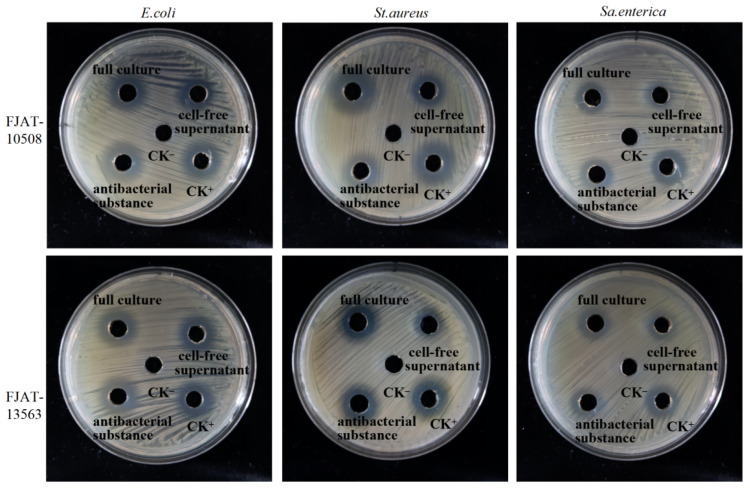
Activity tests evaluation of the antibacterial compounds from FJAT-10508 and FJAT-13563. Notes: The potentially antibacterial compounds were extracted from culture supernatants of the strains FJAT-10508 and FJAT-13563 by the acid (pH 2.0 using 3 M HCl) precipitation method, respectively. Activities of the FJAT-10508 and FJAT-13563 antibacterial compounds against *E. coli*, *St. aureus* and *Sa. enterica* were evaluated using the agar well diffusion method, respectively. An amount of 100 µL of antibacterial compound solution (30 mg·mL^−1^), cell-free supernatant, or full culture was separately loaded in one 9 mm well. Fresh LB broth (CK^−^) and streptomycin sulphate (CK^+^, 200 µg·mL^−1^) were used as the negative and positive controls, respectively.

The BGCs for the potentially antibacterial metabolites were searched in the FJAT-10508 and FJAT-13563 genomes by using antiSMASH 7.0. The results indicated that 12 and 13 BGCs were found in the FJAT-10508 and FJAT-13563 genomes, respectively, and these BGCs were mainly involved in biosynthesis of the antibacterial compounds bacillaene, bacillibactin, bacilysin, butirosin, difffcidin, fengycin, macrolactin, and surfactin (Figure S3). These results inferred that the lipopeptide-like secondary metabolites might be the main antibacterial constituents of FJAT-10508 and FJAT-13563. Accordingly, the potentially antibacterial compounds were extracted by acid precipitation from the cultures of the strains FJAT-10508 and FJAT-13563 and their antibacterial activity against *E. coli*, *St. aureus* and *Sa. enterica* were evaluated using the agar well diffusion method, respectively. As shown in Figure 3, activities of the potentially antibacterial compounds (30 mg·mL^−1^) could comparatively match those of their full cultures and cell-free supernatants of FJAT-10508 and FJAT-13563, except that the activity of the FJAT-10508 antibacterial compound against *St. aureus* was significantly lower than that of its full culture. Therefore, these results demonstrated that the compounds enriched by acid precipitation were one of the contributors to antibacterial activities of the strains FJAT-10508 and FJAT-13563 against *E. coli*, *St. aureus*, and *Sa. enterica*, although the chemical identity of antibacterial compounds needs to be further confirmed by LC-MS/MS with the corresponding standards in the future.

### 3.5. Endospore-Forming Efficiencies of the Strains FJAT-10508 and FJAT-13563

Endospore-forming efficiencies of FJAT-10508 and FJAT-13563 were detected at 24, 48 and 72 h, respectively. The results showed that endospore yields of FJAT-10508 and FJAT-13563 gradually increased with the extension of growth time, and they reached up to 72.41% and 90.83% after 72 h incubation, respectively (Table 1).

### 3.6. Acid and Bile Salt Tolerance of the Strains FJAT-10508 and FJAT-13563

**Figure 4:**
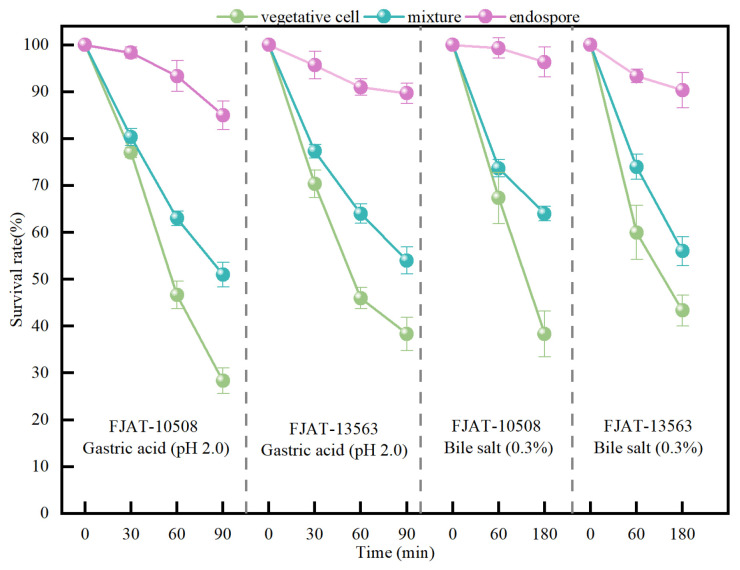
Acid and bile salt tolerance test of the vegetative cells and endospores of FJAT-10508 and FJAT-13563. Notes: For gastric and intestinal tolerance assays, the vegetative cells, mixture of vegetative cells and endospores, and pure endospores of the strains FJAT-10508 and FJAT-13563 were challenged with low pH (pH 2.0) and pepsin in the simulated gastric fluid for 30, 60, and 90 min, and 0.3% (*w*/*v*) bile salts and trypsin in the simulated intestinal fluid for 60 and 180 min, respectively. The working concentration of each vegetative cell, mixture, and pure endospore sample was set as 1 × 10^7^ CFU·mL^−1^ (CFU_initial_). At each time point, the viable CFU value (CFU_viable_) of each sample was determined using the plate colony-counting method. The survival rate (%) was calculated as logCFU_viable_/logCFU_initial_ × 100. All treatments were performed in triplicate independent experiments.

To evaluate gastrointestinal tract tolerance, the vegetative cells, a mixture of vegetative cells and endospores (the ratio of vegetative cell to endospore was approximately 1:1, according to the results of endospore-forming efficiency tests), and pure endospores of the strains FJAT-10508 and FJAT-13563 were challenged with low pH (pH 2.0) and pepsin in the simulated gastric fluid, and with 0.3% (*w*/*v*) bile salt and trypsin in the simulated intestinal fluid, respectively. After exposure to the simulated gastric fluid (pH 2.0) for 90 min, the vegetative cells, mixture, and pure endospores of FJAT-10508 and FJAT-13563 remained viable, with average CFU values of 0.28 × 10^7^ and 0.38 × 10^7^ CFU·mL^−1^, 0.51 × 10^7^ and 0.54 × 10^7^ CFU·mL^−1^, and 0.85 × 10^7^ and 0.9 × 10^7^ CFU·mL^−1^, corresponding to survival rates of 28.33% and 38.33%, 51% and 54%, and 85% and 89.67%, respectively. As for tolerance to 0.3% bile salts, FJAT-10508 and FJAT-13563 retained viable CFU averages of 0.38 × 10^7^ and 0.43 × 10^7^ CFU·mL^−1^, 0.64 × 10^7^ and 0.56 × 10^7^ CFU·mL^−1^, and 0.96 × 10^7^ and 0.9 × 10^7^ CFU·mL^−1^ after exposure to simulated intestinal fluid for 180 min, with survival rates of 38.33% and 43.33%, 64% and 56%, and 96.33% and 90.33%, respectively (Figure 4). These results indicated that the vegetative cells of FJAT-10508 and FJAT-13563 exhibited moderate tolerance to simulated gastrointestinal tract conditions, whereas their endospores demonstrated remarkably high tolerance to these stress environments.

### 3.7. AutoAggregation and Co-Aggregation Abilities of the Strains FJAT-10508 and FJAT-13563

**Figure 5:**
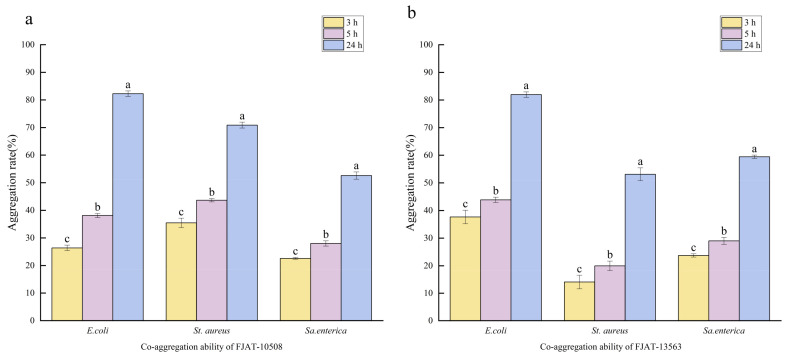
Co-aggregation ability test of the strains FJAT-10508 (**a**) and FJAT-13563 (**b**) with *E. coli*, *St. aureus* and *Sa. enterica*. Notes: For the co-aggregation assays, culture suspensions of each *Bacillus* and pathogen were mixed at a 1:1 (*v*/*v*) ratio, vortexed, and statically co-incubated at 37 °C for 3, 5 and 24 h, with 4 mL of each *Bacillus* or pathogen culture suspension alone as a control treatment. At a certain time, the OD_600nm_ values were measured for the individual suspensions of each *Bacillus* (OD*_Bacillus_*) and each pathogen (OD_pathogen_) before mixing, and for the mixture (OD_mix_). All experiments were conducted with three independent replicates, and the difference lowercase letters indicate a significant difference through the Duncan test (*p* < 0.05).

In autoaggregation assays, the strains FJAT-10508 and FJAT-13563 exhibited autoaggregation rates of 45.28% and 47.99%, respectively, after static incubation for 5 h. Moreover, the autoaggregation rates of FJAT-10508 and FJAT-13563 reached up to 73.62% and 84.93% at 24 h, respectively. The results of co-aggregation assays showed that FJAT-10508 and FJAT-13563 displayed the co-aggregation rates of 82.26% and 81.95%, 70.88% and 53.12%, and 52.60% and 59.45% with *E. coli*, *St. aureus*, and *Sa. enterica* at 24 h, respectively (Figure 5).

### 3.8. Invasion and Cytotoxicity of FJAT-10508 and FJAT-13563 to CACO-2 Cells

The invasion abilities of FJAT-10508 and FJAT-13563 to the CACO-2 cell monolayer were tested using a multiplicity of infection (MOI) of approximately 50:1 (bacteria: mammalian cells). The results showed that the invasion percentages of FJAT-10508 and FJAT-13563 into CACO-2 cells were 0.044 ± 0.008% and 0.037 ± 0.014%, respectively. Furthermore, the cytotoxic effects of the culture supernatants from FJAT-10508 and FJAT-13563 on CACO-2 cells were evaluated using the CCK-8 assay. Cells were treated with PBS (negative control), 1% Triton X-100 (positive control), or bacterial supernatant for 18 h, and cell viability was calculated relative to the PBS-treated control group. The results demonstrated that: (i) treatment with 1% Triton X-100 resulted in near-complete cell death, with a cell viability of 5.8% ± 0.5% (*p* < 0.001); (ii) the negative control (PBS) achieved a cell viability of 99.72 ± 0.91%; (iii) while treatment with FJAT-10508 and FJAT-13563 culture supernatant resulted in the cell viability of 198.71 ± 3.56% and 185.56 ± 4.05%, respectively (Table S6). These results indicated that both FJAT-10508 and FJAT-13563 exhibited no cytotoxic effect on the epithelial cell CACO-2 under the tested conditions.

### 3.9. Antibiotic Susceptibilities of the Strains FJAT-10508 and FJAT-13563

Susceptibilities of the strains FJAT-10508 and FJAT-13563 to twelve antibiotics were determined using the Kirby–Bauer disk diffusion method (Table 2). The results showed that the strains FJAT-10508 and FJAT-13563 exhibited high susceptibility (S) to nine and eight antibiotics, respectively, which included five of the six key antibiotics (chloramphenicol, erythromycin, gentamicin, kanamycin, tetracycline, and streptomycin) as recommended by the European Food Safety Authority (EFSA). However, both strains displayed phenotypic resistance (R) to ampicillin and streptomycin (Table 2).

**Figure 6:**
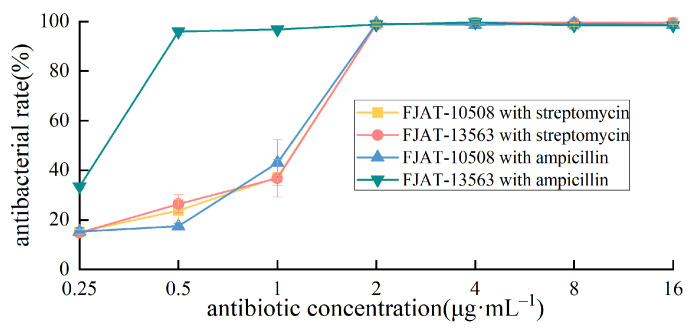
MIC determination of streptomycin and ampicillin on the strains FJAT-10508 and FJAT-13563. Notes: MICs of streptomycin and ampicillin were determined according to “Performance Standards for Antimicrobial Susceptibility Testing, the 45th Edition” of the Clinical and Laboratory Standards Institute (CLSI). All experiments were conducted with three independent replicates.

Accordingly, the minimum inhibitory concentrations (MICs) of ampicillin and streptomycin for strains FJAT-10508 and FJAT-13563 were further determined using the broth microdilution method outlined by CLSI. The results indicated that the MIC values of ampicillin and streptomycin were approximately 2 µg·mL^−1^ and 2 µg·mL^−1^ for FJAT-10508, and 0.5 µg·mL^−1^ and 2 µg·mL^−1^ for FJAT-13563, respectively (Figure 6). These MIC values for both strains were lower than the EFSA-recommended cut-off values of 4 µg·mL^−1^ for ampicillin and 8 µg·mL^−1^ for streptomycin. Collectively, these results indicate that both FJAT-10508 and FJAT-13563 meet the EFSA requirements for antibiotic susceptibility evaluation of probiotic candidates.

### 3.10. Hemolytic Activity of the Strains FJAT-10508 and FJAT-13563

**Figure 7:**
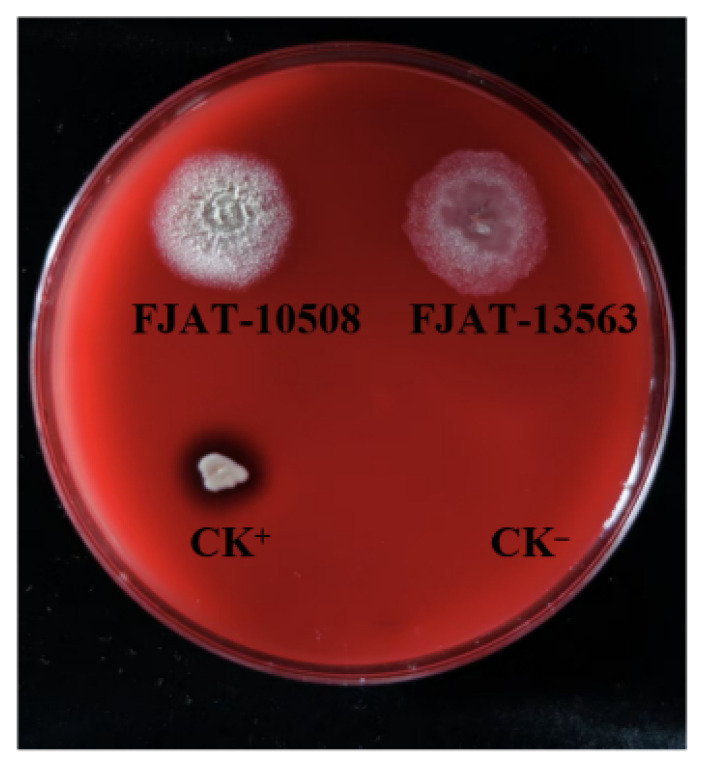
Hemolytic activity detection of the strains FJAT-10508 and FJAT-13563. Notes: The hemolytic activity of the strains FJAT-10508 and FJAT-13563 was evaluated using the Columbia blood agar plate, and *St. aureus* and fresh LB broth were used as positive (CK^+^) and negative (CK^−^) controls, respectively.

The hemolytic activities of the strains FJAT-10508 and FJAT-13563 were assessed using 5% sheep blood agar. As shown in Figure 7, the positive control strain *Staphylococcus aureus* FJAT-2450 exhibited a clear hemolysis zone around the colony, indicating typical β-hemolysis associated with pathogenicity. The strains FJAT-10508 and FJAT-13563 were both α-hemolysis, being considered safe.

## 4. Discussions

The systematic isolation and screening of *Bacillus* strains that combine robust enzymatic activity with potent antibacterial ability against prevalent animal pathogens is a pivotal research priority for the development of highly effective feed probiotics [49]. In this study, a total of 394 *Bacillus* strains underwent the measurement of extracellular-enzyme producing abilities and antibacterial activities against *E. coli*, *St. aureus* and *Sa. enterica* by using the transparent ring method and the double-layer agar method, respectively. Our results indicated that these *Bacillus* strains exhibited good extracellular-enzyme-producing abilities, but relatively poor antibacterial activities against *E. coli*, *St. aureus* and *Sa. enterica* under the conditions of this study. There were 203, 305, and 158 *Bacillus* strains with producing abilities of extracellular cellulose (average D/d ratio value range of 1.33–6.9), protease (1.09–3.5) and amylase (1.12–2.45), but just 20, 7, and 5 *Bacillus* strains with antibacterial activities against *E. coli* (average inhibition zone diameter range of 11.35–19.84 mm), *St. aureus* (12.32–16.76 mm), and *Sa. enterica* (11.32–17.1 mm), respectively. In addition, 128 *Bacillus* strains could produce all three extracellular digestive enzymes, but only FJAT-10508 and FJAT-13563 exhibited simultaneously inhibitory activities against three pathogens. According to their comprehensive assessment of extracellular enzyme-producing abilities and antibacterial activities against *E. coli*, *St. aureus*, and *Sa. enterica*, the strains *B. velezensis* FJAT-10508 and FJAT-13563 were therefore selected to further evaluate systematically probiotic properties in vitro, which include endospore-forming efficiencies, antibiotic susceptibilities, hemolytic activities, prediction of putative virulence factors and ARGs, autoaggregation and co-aggregation abilities, and tolerance to bile salts and simulated gastrointestinal fluids.

A key technological advantage of *Bacillus*-based products is their endospore-forming ability, which confers high stability during long term storage and feed processing operations such as conditioning and pelleting [15]. Our results showed that endospore yields of *B. velezensis* FJAT-10508 and FJAT-13563 could reach up to 72.41% and 90.83% after 72 h incubation, respectively, being consistent with previous reports for other *B. velezensis* strains that more than 50% of sporulation rate could routinely achieve within 72 h [50,51]. These high-endospore titers of FJAT-10508 and FJAT-13563 would bring the potential for robust survival in adverse circumstances, such as the gastrointestinal environment and feed pelleting. Additionally, the results of our preliminary thermotolerance tests revealed that exposure to 80 °C for 20 min had no significantly deleterious effect on endospore viability of FJAT-10508 and FJAT-13563, and their endospores remained more than 30% of survival rates under another 5 min challenge at 100 °C, indicating satisfactory heat resistance.

Tolerance to gastric acidity and intestinal bile salts is a prerequisite for probiotic survival in the gastrointestinal tract of animals [52]. In this study, the vegetative cells, a mixture of vegetative cells and endospores at a ratio of approximately 1:1, and pure endospores of the strains FJAT-10508 and FJAT-13563 were used to comparatively assess their tolerance capacity to the simulated gastric fluid (pH 2.0 and pepsin) and simulated intestinal fluid (0.3% bile salt and trypsin), respectively. Our results demonstrated that the survival rates of FJAT-10508 and FJAT-13563 gradually increased from 28.33–38.33% for vegetative cells to 51–54% for mixture and to 85–89.67% for pure endospores under the stress of pH 2.0 for 90 min, and from 38.33–43.33% for vegetative cells to 56–64% for mixture and to 90.33–96.33% for pure endospores under the stress of 0.3% bile salt for 180 min, respectively. These results indicated that the endospores of FJAT-10508 and FJAT-13563 had significantly greater stress tolerance in gastrointestinal environments than their vegetative cells. At present, *Bacillus*-based probiotics are mainly formulated for delivery as endospores, but the ability of endospores to germinate in the gut remains debatable [53]. To optimize their application, cells should be delivered in their vegetative state, but the sensitivity of *Bacillus* vegetative cells to gastric acidity and intestinal bile salts likely prevents this. Therefore, exploring new formulations of *Bacillus*-based probiotics, such as mixtures of vegetative cells and endospores, is of great significance.

The capacity for autoaggregation and co-aggregation is considered a prerequisite for *Bacillus* spp. to colonize and competitively inhibit the adhesion of the corresponding pathogens to the gastrointestinal tract of host animals [54]. The results of autoaggregation assays demonstrated that the autoaggregation rates of the strains FJAT-10508 and FJAT-13563 could reach to 45.28% and 47.99% at 5 h, being very close to the autoaggregation threshold of 50% defined for highly adhesive isolates [55], and to 73.62% and 84.93% after static incubation for 24 h, respectively. Moreover, the results showed that both FJAT-10508 and FJAT-13563 also exhibited the remarkable co-aggregation capacities with *E. coli* (82.26% and 81.95%), *St. aureus* (70.88% and 53.12%), and *Sa. enterica* (52.60% and 59.45%) at 24 h, respectively, being consistent with the findings of Blibech et al. [56]. Notably, in addition to physical co-aggregation, both strains demonstrated strong antagonistic activity against these pathogens through the production of diffusible antimicrobial compounds (as evidenced by inhibition zone assays), suggesting a dual mechanism of action combining growth inhibition and co-aggregation. This combined effect of antimicrobial substance production and co-aggregation ability provides a competitive advantage to the *Bacillus* strains, enabling them not only to inhibit pathogens but also to physically eliminate them from the environment, thereby offering a dual-layer of protection [54]. Therefore, these results would confer FJAT-10508 and FJAT-13563 promising probiotic potentials.

Safety validation of probiotic candidates is an essential prerequisite for their commercial applications in animal farming. First of all, susceptibility to clinically relevant antimicrobials is a key concern, as the presence of acquired resistance determinants in ARB can promote horizontal gene transfer of ARGs to other microorganisms within the animal gut and husbandry environments [57]. Therefore, susceptibilities of the strains FJAT-10508 and FJAT-13563 to 12 antibiotics, which include the six key antibiotics (chloramphenicol, erythromycin, gentamicin, kanamycin, tetracycline, and streptomycin) as recommended by EFSA, were determined by the Kirby–Bauer disk diffusion method under the guidance of CLSI. The results demonstrated that the strains FJAT-10508 and FJAT-13563 were highly susceptible (S) and intermediately sensitive (I) to 9 and 1, and 8 and 2 antibiotics, respectively. However, the strains FJAT-10508 and FJAT-13563 exhibited resistance (R) to ampicillin and streptomycin. Accordingly, the MICs of ampicillin and streptomycin on the strains FJAT-10508 and FJAT-13563 were further determined according to the guidelines of CLSI. Fortunately, the results showed that the MIC values of ampicillin and streptomycin on both strains were ≤2 µg·mL^−1^, being lower than the relevant MIC cut-off values of 4 µg·mL^−1^ for ampicillin and 8 µg·mL^−1^ for streptomycin as recommended by EFSA. Taken together, both FJAT-10508 and FJAT-13563 could meet the requirements for antibiotic susceptibility testing of probiotic candidates. Meanwhile, putative ARGs and the mobile genetic elements flanking them in the FJAT-10508 and FJAT-13563 genomes were further predicted using the CARD and Mobilome Annotation Pipeline, respectively. The results showed that 160 and 148 putative ARG-related genes, which were associated with 38 categories of antibiotics (such as aminoglycoside, cephalosporin, fluoroquinolone, glycopeptide, macrolide, rifamycin, and tetracycline) were detected in the FJAT-10508 and FJAT-13563 genomes, respectively. Moreover, the FJAT-10508 and FJAT-13563 genomes could be identified as having only one kind of mobile genetic elements (compositional outlier region) around 24 and 15 putative ARGs in, respectively, without any common mobile genetic elements (such as plasmids, prophages, insertion sequences, inverted repeat elements).

On the other hand, the results of hemolytic activity tests using 5% sheep blood agar indicated that both FJAT-10508 and FJAT-13563 exhibited the phenotype of α-hemolysis, which is considered to be safe [58,59]. Moreover, the lack of the hemolysin-related genes, such as *hblA*, *hblC*, and *hblD* found in the highly pathogenic *Bacillus cereus* [60], was further confirmed by whole-genomic search. Meanwhile, putative virulence-related genes and the mobile genetic elements flanking them were predicted in the FJAT-10508 and FJAT-13563 genomes. The results showed that 226 and 231 putative virulence genes were predicted in the FJAT-10508 and FJAT-13563 genomes, respectively, which are mainly involved in the functions of adherence, effector delivery system, exoenzyme, exotoxin, immune modulation, invasion, and stress survival. Moreover, only 19 and 13 putative virulence genes in the FJAT-10508 and FJAT-13563 genomes were accompanied by a single mobile genetic element, the compositional outlier region, respectively, without any common mobile genetic elements. Importantly, a pangenome analysis of 435 *B. velezensis* genomes was performed to evaluate whether the putative virulence genes are typical, low-risk housekeeping genes, or high-risk features. The results demonstrated that the putative virulence genes via VFDB annotations of FJAT-10508 and FJAT-13563 exhibited the pangenome categories of 56.34% and 57.95% cloud genes, 16.9% and 17.33% core genes, 10.42% and 8.52% shell genes, 6.2% and 5.97% soft core genes, and 10.14% and 10.23% strict core genes, respectively. Furthermore, the potential high-risk virulence factors of FJAT-10508 and FJAT-13563, such as exoenzyme (66.7% cloud genes), adherence factors (64.3% cloud), motility (61.7% cloud), and effector delivery systems (59.3% cloud), were predominantly strain-specific and not conserved across the species *B. velezensis*, suggesting an absence of genomic hallmarks of pathogenic bacteria at the species-level. These results indicated that most of the predicted virulence genes from the FJAT-10508 and FJAT-13563 genomes were not conserved pathogenic determinants, but just bestowed strain-specific accessory functions. Consistent with these genomic findings, the CCK-8 cytotoxicity assay demonstrated that the culture supernatant of *B. velezensis* FJAT-10508 and FJAT-13563 did not exert cytotoxic effects on CACO-2 cells. Moreover, these findings aligned with previous safety evaluations of the species *B. velezensis*, as well as other *Bacillus* species, which have shown no cytotoxicity to intestinal epithelial cells [61]. According to Pournejati et al. [62], values exceeding 100% in CCK-8 assays should be interpreted as an absence of cytotoxicity rather than as a technical error, as such values may reflect the presence of growth-promoting metabolites in the test sample. Meanwhile, the results of the invasion assays indicated that both FJAT-10508 and FJAT-13563 were non-invasive and thus unable to cause invasive disease [63]. Taken together, these results suggested that the strains FJAT-10508 and FJAT-13563 had no appreciable threat for feed use in animal farming, although needing a key in vivo safety evaluation in the future.

## 5. Conclusions

In this study, the strains *B. velezensis* FJAT-10508 and FJAT-13563 were screened from 394 *Bacillus* strains based on their comprehensive performance in extracellular enzyme production and antibacterial activities against *E. coli*, *St. aureus*, and *Sa. enterica*. Moreover, the compounds enriched by acid precipitation were identified as one of the contributors for antibacterial activities of FJAT-10508 and FJAT-13563. According to in vitro probiotic potential assessments and whole-genome analyses, both FJAT-10508 and FJAT-13563 exhibited α-hemolysis, which is considered safe, lacked genomic hallmarks of pathogenic bacteria, and had no invasion or cytotoxicity effects on CACO-2 cells. In addition, they met the requirements of the antibiotic susceptibility tests recommended by EFSA for probiotic candidates and exhibited excellent endospore-forming efficiency, good autoaggregation and coaggregation abilities, and strong tolerance to simulated gastrointestinal tract environments. Therefore, the strains *B. velezensis* FJAT-10508 and FJAT-13563 demonstrated desirable in vitro probiotic properties. To accelerate their practical application, key aspects such as in vivo efficacy and safety, host response, and ecological impact in animal farming systems should be further evaluated through large-scale animal feeding trials in future studies.

## Figures and Tables

**Table 1:** Endospore-forming efficiencies of the strains FJAT-10508 and FJAT-13563 at different growth periods [log/(CFU mL^−1^)].

Strain		24 h			48 h			72 h	
	Total Viable Cells	Endospores	Yield	Total Viable Cells	Endospores	Yield	Total Viable Cells	Endospores	Yield
FJAT-10508	7.29 ± 0.05 b	6.58 ± 0.16 b	13.00%	7.75 ± 0.02 b	7.46 ± 0.04 b	52.07%	8.27 ± 0.10 a	8.14 ± 0.05 a	72.41%
FJAT-13563	7.99 ± 0.16 b	7.10 ± 0.16 a	13.10%	8.58 ± 1.08 a	8.30 ± 0.32 b	52.49%	8.56 ± 0.25 a	8.52 ± 0.06 c	90.83%

Notes: The cultures were harvested at 24 h, 48 h, and 72 h, and divided into two portions, respectively. One was heated at 80 °C for 20 min, the other was not. The endospore-forming efficiency (%) was calculated as CFU_heated_/CFU_non-heated_ × 100. Within each row, values with different lowercase letter are significantly different (*p* < 0.05).

**Table 2:** Antibiotic sensitivity test of the strains FJAT-10508 and FJAT-13563.

Antibiotic	Content per disc/μg	Judgement Standard/mm ^1^			FJAT-10508		FJAT-13563	
		Drug Tolerance (R)	Inhibition (I)	High Sensitivity (S)	Inhibition Zone		Inhibition Zone	
					Diameter/mm ^2^	Sensitivity	Diameter/mm	Sensitivity
Polymyxin B	300	≤8	8–11	≥12	13.61 ± 0.33	S	11.21 ± 0.01	I
Sulfanilamide	300	≤12	13–16	≥17	16.81 ± 5.95	S	15.28 ± 2.14	I
Enrofloxacin	10	≤22	23–27	≥28	25.73 ± 0.86	S	26.12 ± 1.24	S
Chloramphenicol	30	≤12	13–17	≥18	27.02 ± 0.68	S	23.6 ± 2.45	S
Erythromycin	15	≤13	14–22	≥23	27.45 ± 0.61	S	28.22 ± 3.49	S
Ampicillin	10	≤13	14–16	≥17	9.81 ± 1.51	R	13.86 ± 0.05	R
Gentamicin	10	≤12	13–14	≥15	21.72 ± 0.51	S	17.2 ± 1.53	S
Cefaclor	30	≤14	15–17	≥18	38.11 ± 0.24	S	27.8 ± 6.99	S
Tetracycline	30	≤14	15–18	≥19	14.88 ± 0.44	I	22.73 ± 1.82	S
Neomycin	30	≤12	13–16	≥17	17.32 ± 3.87	S	17.6 ± 0.18	S
Streptomycin	10	≤11	12–14	≥15	11 ± 6.04	R	9.84 ± 0.62	R
Kanamycin	30	≤13	14–17	≥18	26.38 ± 1.42	S	23.14 ± 2.77	S

Notes: ^1^ The breakpoint criteria in antibiotic susceptibility testing were defined according to “Performance Standards for Antimicrobial Susceptibility Testing, the 45th Edition” of the Clinical and Laboratory Standards Institute (CLSI). ^2^ All experiments were conducted with three independent replicates. Data were presented as the mean ± standard deviation.

## Data Availability

The original contributions presented in this study are included in the article/Supplementary Material. Further inquiries can be directed to the corresponding authors.

## Supplementary Materials

The following supporting information can be downloaded at: https://www.mdpi.com/article/10.3390/microorganisms14040834/s1, Supplementary Figure S1: Circular genomic map of the strains *B. velezensis* FJAT-10508 and FJAT-13563. Notes: The genome of the strain FJAT-57093 comprised a single circular chromosome of 3,452,556 bp, with an average G+C content of 46.49% and no detectable plasmid. A total of 3730 protein-coding genes were predicted. From outside to inside, the rings represent: Ring 1, Genome scale; Rings 2 and 3, CDS on the positive and negative strands colored by COG functional categories; Ring 4, rRNA and tRNA; Ring 5, ncRNA, prophage, genomic islands (GI), and insertion sequences (IS); Ring 6, GC content. Outward red peaks indicate regions where GC content is higher than the genomic average, with peak height reflecting the degree of deviation; inward blue peaks indicate regions where GC content is lower than the average; Ring 7: GC-skew calculated as (G−C)/(G+C), generally, positive skew (>0) corresponds to the leading strand, while negative skew (<0) corresponds to the lagging strand. Supplementary Figure S2: Mapping virulence genes of FJAT-10508 and FJAT-13563 to pangenome categories of the species *B. velezensis.* Notes: To evaluate whether the putative virulence factors are typical, low-risk housekeeping, or high-risk features, a pangenome analysis of 434 *B. velezensis* genomes (including all NCBI complete genomes and FJAT 57093) were performed using PGAP2 (https://github.com/bucongfan/PGAP2) (Bu et al., 2025 [41]) to map virulence genes of FJAT 57093 to pangenome categories. Supplementary Figure S3: Comparative atlas of the biosynthetic gene clusters for the potentially antibacterial metabolites in the strains FJAT-10508 and FJAT-13563. Notes: The BGCs for the potentially antibacterial metabolites were searched in the FJAT-10508 and FJAT-13563 genomes by using antiSMASH 7.0. Colored arrows represent the predicted genes and their transcriptional orientation; identical colors indicate the orthologous pairs. Cluster numbers are indicated below the respective regions. Gray arrows denote the strain-specific genes, and blank spaces indicate absent or rearranged segments. Table S1: Probiotic candidate screening of from 394 *Bacillus*-like strains based on their extracellular-enzyme production abilities and antibacterial activities against *E. coli*, *St. aureus* and *Sa. enterica*. Table S2: Putative virulence genes predicted in the FJAT-10508 and FJAT-13563 genomes. Table S3: Putative ARGs predicted in the FJAT-10508 and FJAT-13563 genomes. Table S4: Statistics of transferable elements carrying virulence and resistance genes in the FJAT-10508 and FJAT-13563 genomes. Table S5: Pangenome categories of the 435 *B. elezensis* genomes. Table S6: Cytotoxicity assays of the FJAT-10508 and FJAT-13563 culture supernatants to the Caco-2 cells.
