# Supplementary material for: Screening and Evaluation In Vitro of Bacillus-Based Probiotics for Feed Additives
Source: Microorganisms. 2026 Apr 7;14(4):834. doi: 10.3390/microorganisms14040834 (PMC13119242; doi:10.3390/microorganisms14040834)
Supplement: Supplementary file 1 [file microorganisms-14-00834-s001.zip › microorganisms-4134370-supplementary.pdf]

Screening and Evaluation *in vitro* of *Bacillus*-based Probiotics for Feed Additives

Yujun Mao [1,2], Xiaofang Lou [2,3], Jianmei Che [2], Xiaoyun Huang [2], Yanping Chen [2], Jianglin Lan [2], Meichun Chen [2],
Xin Liu [2], Qinlou Huang [2], Xiusheng Huang [2,*] and Jieping Wang [2,*]

[1] College of Life Sciences, Fujian Agriculture and Forestry University, Fuzhou 350002, China;
yujunmao2026@163.com

[2] Fujian Engineering and Technology Research Center for Recycling Agriculture in Hilly Areas, Institute of Resources, Environment and Soil Fertilizer, Fujian Academy of Agricultural Sciences, Fuzhou 350003, China; 18208447534@163.com (X.L.); chejm2002@163.com (J.C.);
huangxy364@163.com (X.H.); chenyanping@faas.cn (Y.C.); lanfz2008@163.com (J.L.); cmczjw@163.com (M.C.); fzliuxin@yeah.net (X.L.); hql202@126.com (Q.H.)

[3] College of Animal Science, Fujian Agriculture and Forestry University, Fuzhou 350002, China

* Correspondence: hxs706@163.com (X.H.); wangjieping2011@163.com (J.W.)

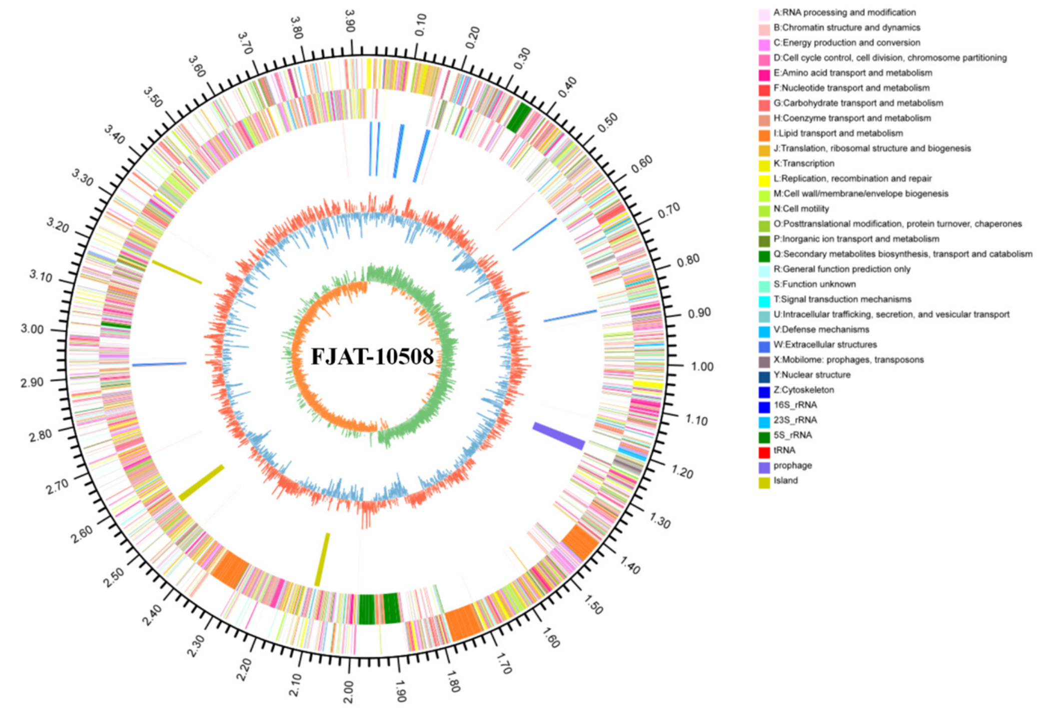

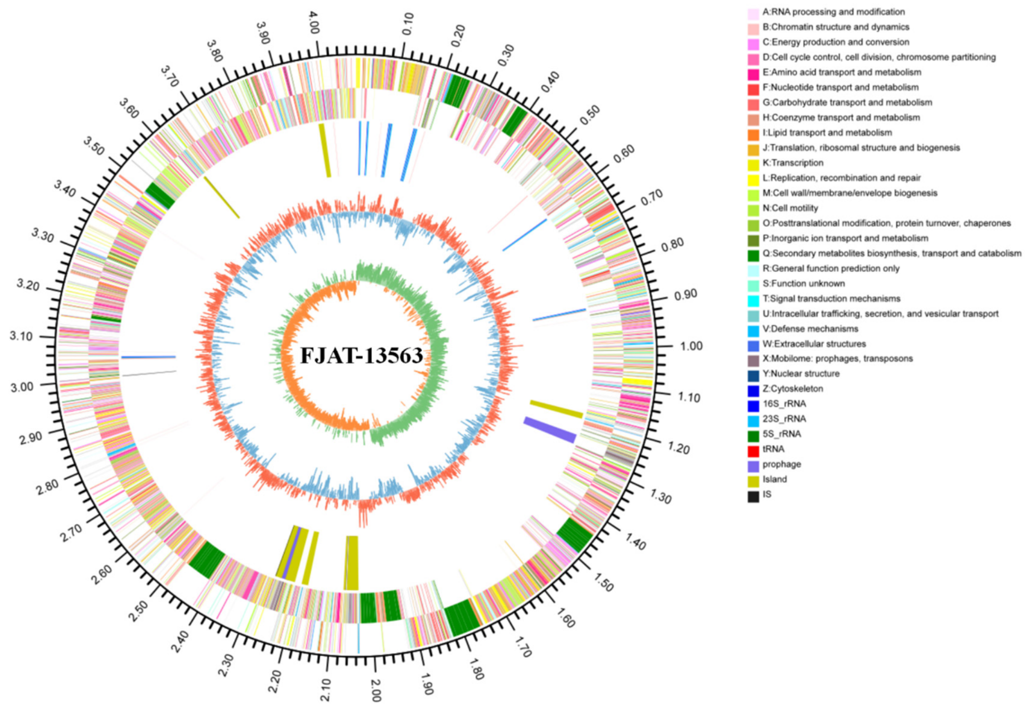

Supplementary Figure S1. Circular genomic map of the strains *B. velezensis* FJAT-10508 and FJAT-13563

Notes: The genome of the strain FJAT-57093 comprised a single circular chromosome of 3,452,556 bp, with an average G+C content of 46.49% and no detectable plasmid. A total of 3,730 protein-coding genes were predicted. From outside to inside, the rings represent: Ring 1, Genome scale; Rings 2 and 3, CDS on the positive and negative strands colored by COG functional categories; Ring 4, rRNA and tRNA; Ring 5, ncRNA, prophage, genomic islands (GI), and insertion sequences (IS); Ring 6, GC content. Outward red peaks indicate regions where GC content is higher than the genomic average, with peak height reflecting the degree of deviation; inward blue peaks indicate regions where GC content is lower than the average; Ring 7: GC-skew calculated as (G-C)/(G+C), generally, positive skew (>0) corresponds to the leading strand, while negative skew (<0) corresponds to the lagging strand.

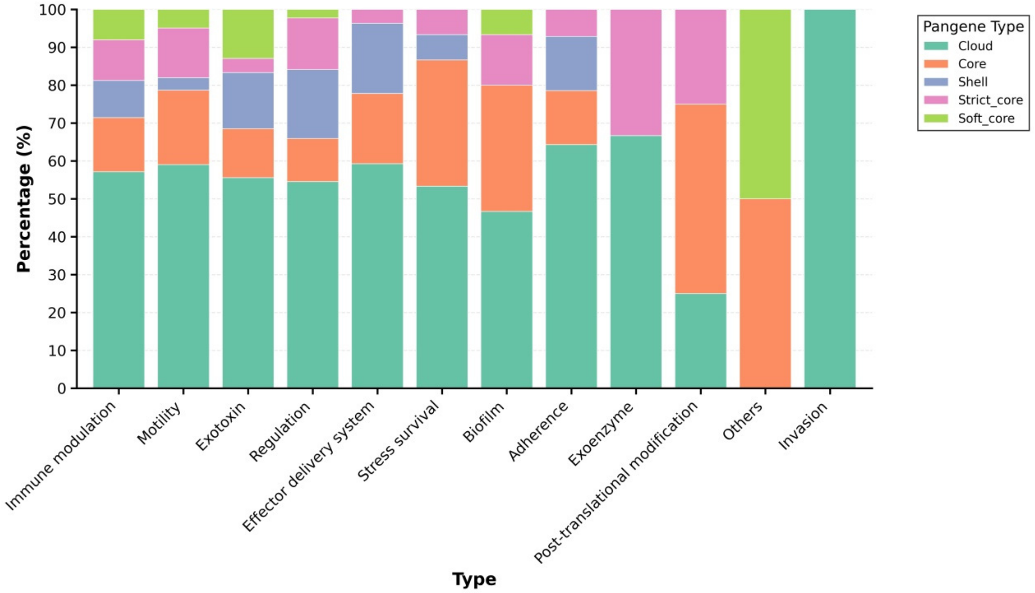

FJAT-10508

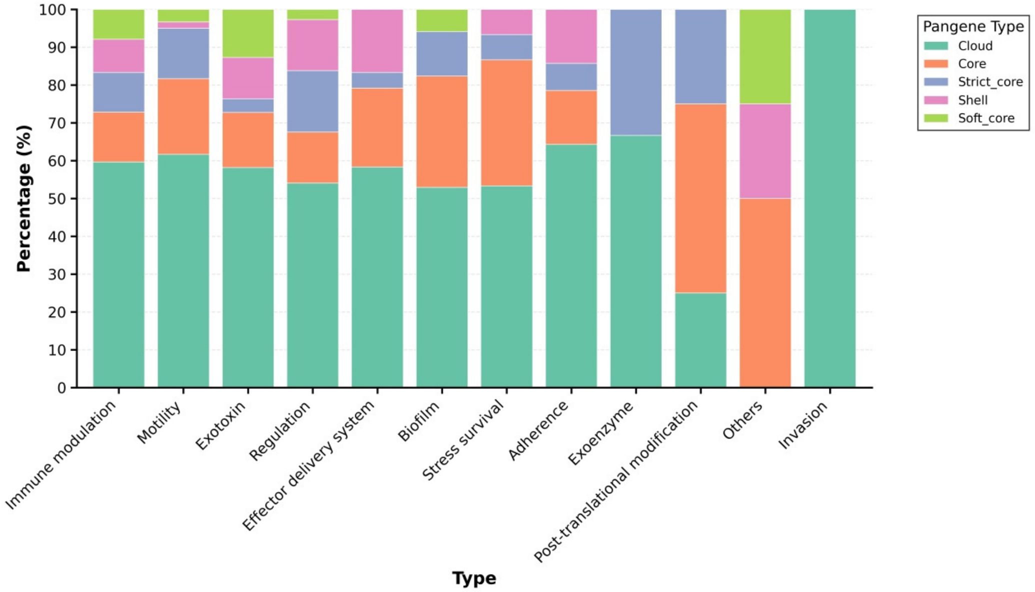

FJAT-13563

Supplementary Figure S2. Mapping virulence genes of FJAT-10508 and FJAT-13563 to pangenome categories of the species *B. velezensis*

Notes: To evaluate whether the putative virulence factors are typical, low-risk housekeeping, or high-risk features, a pangenome analysis of 434 *B. velezensis* genomes (including all NCBI complete genomes and FJAT 57093) were performed using PGAP2 (https://github.com/bucongfan/PGAP2) (Bu et al., 2025 [41]) to map virulence genes of FJAT 57093 to pangenome categories.

Bu CF, Zhang H, Zhang FN, Liang WH, Gao H, Zhao J, Lv FM, Xue RK, Liu Q, Zhang ZW, Jin Z, Xiao JF. PGAP2: A comprehensive toolkit for prokaryotic pan-genome analysis based on fine-grained feature networks. Nat Commun, 2025, 16(1):9865.

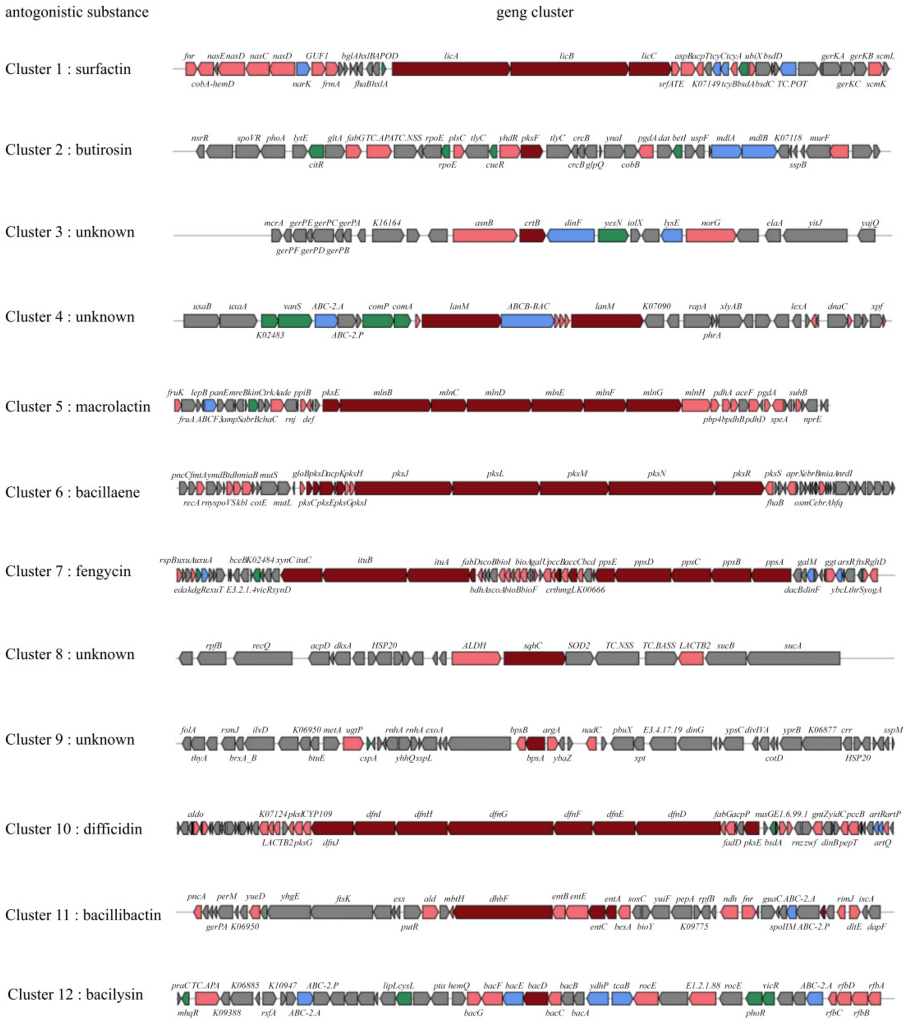
antogonistic substance
geng cluster
Cluster 1 : surfactin
Cluster 2 : butirosin
Cluster 3 : unknown
Cluster 4 : unknown
Cluster 5 : macrolactin
Cluster 6 : bacillaene
Cluster 7 : fengycin
Cluster 8 : unknown
Cluster 9 : unknown
Cluster 10 : difficidin
Cluster 11 : bacillibactin
Cluster 12 : bacilysin

FJAT-10508

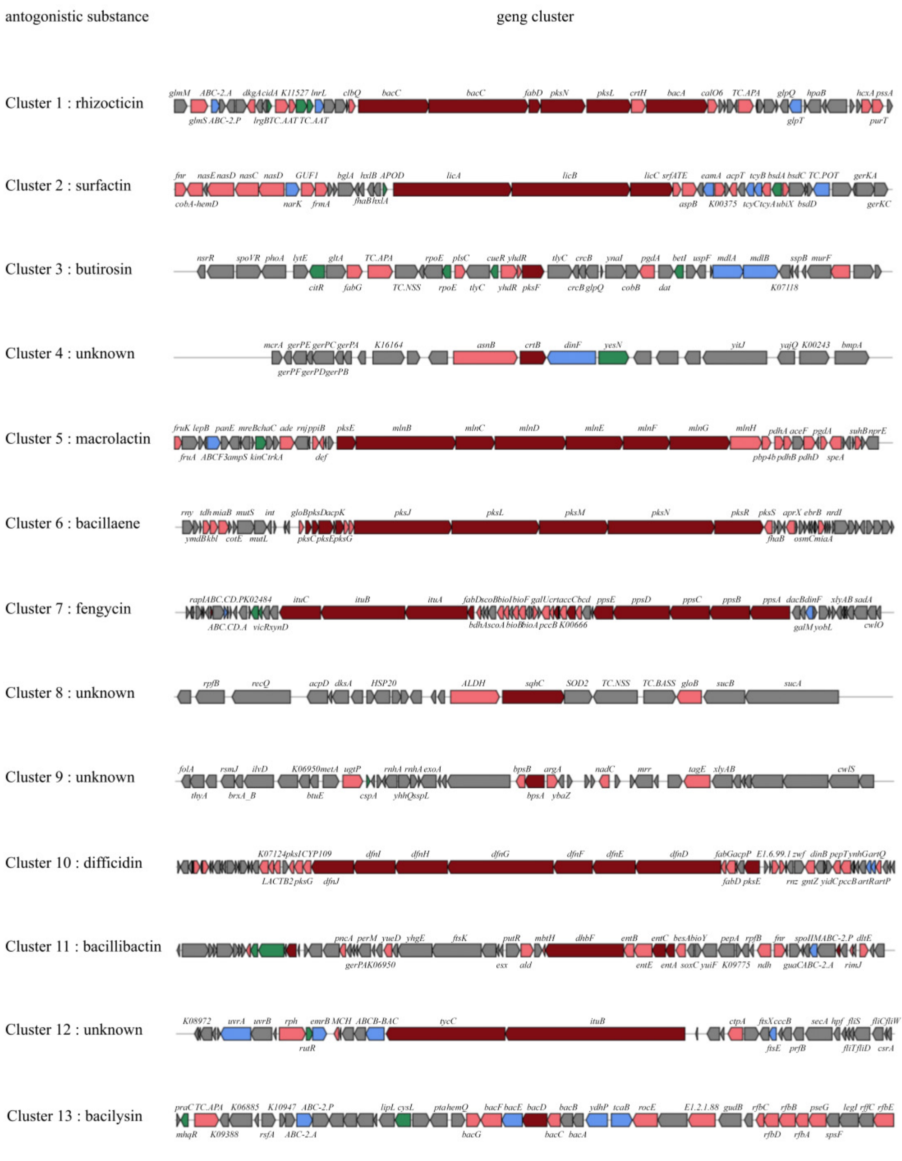

FJAT-13563

Supplementary Figure S3. Comparative atlas of the biosynthetic gene clusters for the potentially antibacterial metabolites in the strains FJAT-10508 and FJAT-13563. Notes: The BGCs for the potentially antibacterial metabolites were searched in the FJAT-10508 and FJAT-13563 genomes by using antiSMASH 7.0. Colored arrows represent the predicted genes and their transcriptional orientation; identical colors indicate the orthologous pairs. Cluster numbers are indicated below the respective regions. Gray arrows denote the strain-specific genes, and blank spaces indicate absent or rearranged segments.

Table S1 Probiotic candidate screening of from 394 *Bacillus*-like strains based on their extracellular-enzyme production abilities and antibacterial activities against *E. coli*, *St. aureus* and *Sa. enterica*

No.	Strain No.[#]	Species Name[*]	Cellulase	Protease	Amylase	*E. coli*	*St. aureus*	*Sa. enterica*
1	FJAT-202	*Bacillus velezensis*	1.88±0.01	1.45±0.05	1.67±0.11	nd	13.44±0.35	nd
2	FJAT-277	*Bacillus licheniformis*	2.3±0.06	1.22±0.02	1.56±0.36	nd	nd	nd

3	FJAT-278	*Bacillus licheniformis*	2.7±0.46	2.07±0.2	1.95±0.3	nd	nd	nd
4	FJAT-361	*Bacillus pumilus*	6.9±1.02	1.83±0.07	2.29±0.04	nd	nd	nd
5	FJAT-362	*Bacillus pumilus*	2.55±0.44	2.29±0.5	nd	nd	nd	nd
6	FJAT-363	*Bacillus pumilus*	nd	nd	nd	nd	nd	nd
7	FJAT-401	*Bacillus pumilus*	nd	2.06±0.13	nd	14.78±0.05	nd	nd
8	FJAT-421	*Bacillus pumilus*	nd	1.94±0.06	nd	15.31±0.13	nd	nd
9	FJAT-512	*Bacillus pumilus*	nd	1.87±0.14	nd	nd	nd	nd
10	FJAT-515	*Bacillus pumilus*	2.81±0.31	nd	1.6±0.2	nd	nd	nd
11	FJAT-705	*Bacillus pumilus*	nd	1.52±0.08	nd	nd	nd	nd
12	FJAT-924	*Bacillus pumilus*	nd	nd	nd	nd	nd	nd
13	FJAT-4400	*Bacillus pumilus*	nd	2.67±0.5	nd	14.22±0.34	nd	nd
14	FJAT-4415	*Bacillus licheniformis*	2.58±0.05	1.46±0.17	nd	nd	nd	nd
15	FJAT-4479	*Bacillus pumilus*	nd	2.37±0.06	nd	nd	nd	nd
16	FJAT-4500	*Bacillus pumilus*	nd	2.09±0.04	nd	nd	nd	nd
17	FJAT-4563	*Bacillus pumilus*	nd	1.83±0.14	1.69±0.03	nd	nd	nd
18	FJAT-4605	*Bacillus licheniformis*	nd	2.21±0.17	nd	nd	nd	nd
19	FJAT-4648	*Bacillus licheniformis*	nd	2.15±0.12	nd	nd	nd	nd
20	FJAT-5563	*Bacillus licheniformis*	nd	nd	nd	nd	nd	nd
21	FJAT-5665	*Bacillus pumilus*	nd	nd	nd	nd	nd	nd
22	FJAT-7267	*Bacillus licheniformis*	nd	2.31±0.28	nd	nd	nd	nd
23	FJAT-7269	*Bacillus pumilus*	2.3±0.23	2.07±0.26	2.88±0.88	nd	nd	nd
24	FJAT-8350	*Bacillus licheniformis*	nd	1.81±0.13	nd	nd	nd	nd
25	FJAT-8771	*Bacillus pumilus*	nd	2.62±0.03	nd	nd	nd	nd
26	FJAT-8779	*Bacillus pumilus*	nd	1.61±0.05	nd	18.24±0.21	nd	nd
27	FJAT-8853	*Bacillus licheniformis*	nd	nd	1.16±0.02	nd	nd	nd
28	FJAT-10503	*Bacillus velezensis*	nd	nd	nd	nd	nd	nd
29	FJAT-10508	*Bacillus pumilus*	1.92±0.13	1.5±0.16	1.75±0.13	16.02±0.18	16.76±0.56	12.6±0.58
30	FJAT-10515	*Bacillus pumilus*	nd	1.79±0.03	nd	nd	nd	nd
31	FJAT-10518	*Bacillus pumilus*	nd	2.13±0.35	nd	nd	nd	nd
32	FJAT-10670	*Bacillus licheniformis*	nd	1.65±0.09	nd	nd	nd	nd
33	FJAT-10671	*Bacillus pumilus*	nd	nd	nd	nd	nd	nd
34	FJAT-10695	*Bacillus licheniformis*	nd	2.42±0.47	nd	14.56±0.15	nd	nd
35	FJAT-10867	*Bacillus licheniformis*	2.1±0.33	2.59±0.5	nd	nd	nd	nd
36	FJAT-10960	*Bacillus pumilus*	2.03±0.01	1.76±0.31	1.34±0.01	nd	nd	nd
37	FJAT-10964	*Bacillus licheniformis*	nd	2.2±0.18	nd	nd	nd	nd
38	FJAT-10984	*Bacillus licheniformis*	2.02±0.32	1.98±0.34	2.22±0.4	nd	nd	nd
39	FJAT-10991	*Bacillus licheniformis*	2.8±0.31	nd	1.69±0.17	nd	nd	nd
40	FJAT-11358	*Bacillus licheniformis*	nd	nd	nd	nd	nd	nd
41	FJAT-11675	*Bacillus licheniformis*	2.3±0.1	1.25±0.06	1.5±0.17	nd	nd	nd
42	FJAT-11689	*Bacillus licheniformis*	3.3±1.52	1.25±0.08	1.25±0.02	nd	nd	nd
43	FJAT-11690	*Bacillus licheniformis*	2.5±0.23	nd	1.29±0.04	nd	nd	nd
44	FJAT-12271	*Bacillus licheniformis*	nd	nd	nd	nd	nd	nd
45	FJAT-13425	*Bacillus pumilus*	nd	2.26±0.14	nd	nd	nd	nd
46	FJAT-13429	*Bacillus pumilus*	nd	1.7±0.03	nd	nd	nd	nd
47	FJAT-13430	*Bacillus pumilus*	nd	1.75±0.1	nd	nd	nd	nd
48	FJAT-13436	*Bacillus pumilus*	nd	2.45±0.02	nd	nd	nd	nd
49	FJAT-13517	*Bacillus pumilus*	nd	1.85±0.07	nd	nd	nd	nd
50	FJAT-13538	*Bacillus pumilus*	nd	1.95±0.17	nd	nd	nd	nd
51	FJAT-13544	*Bacillus pumilus*	nd	2.37±0.89	nd	nd	nd	nd
52	FJAT-13545	*Bacillus pumilus*	nd	1.97±0.03	nd	nd	nd	nd

53	FJAT-13556	*Bacillus velezensis*	nd	1.64±0.09	nd	15.90±0.16	nd	nd
54	FJAT-13563	*Bacillus pumilus*	2.13±0.13	1.91±0.08	1.56±0.01	14.36±2.47	13.96±0.94	11.32±0.24
55	FJAT-13565	*Bacillus pumilus*	nd	1.71±0.04	nd	nd	nd	nd
56	FJAT-13595	*Bacillus licheniformis*	nd	2.2±0.08	nd	15.42±0.38	nd	nd
57	FJAT-13825	*Bacillus pumilus*	1.84±0.29	1.96±0.08	nd	nd	nd	nd
58	FJAT-13835	*Bacillus pumilus*	nd	1.8±0.21	nd	14.10±0.23	nd	nd
59	FJAT-13848	*Bacillus pumilus*	2.32±0.58	1.61±0.07	1.65±0.17	nd	nd	nd
60	FJAT-14069	*Bacillus licheniformis*	nd	1.7±0.03	nd	nd	nd	nd
61	FJAT-14071	*Bacillus pumilus*	2.23±0.11	1.61±0.19	2.24±0.21	nd	nd	nd
62	FJAT-14076	*Bacillus pumilus*	nd	nd	nd	nd	nd	nd
63	FJAT-14077	*Bacillus licheniformis*	nd	nd	nd	nd	nd	nd
64	FJAT-14084	*Bacillus pumilus*	2.38±0.78	1.38±0.05	1.25±0.06	nd	nd	nd
65	FJAT-14135	*Bacillus licheniformis*	nd	1.91±0.01	nd	nd	nd	nd
66	FJAT-14169	*Bacillus pumilus*	nd	2.65±0.15	2.21±0.21	nd	nd	nd
67	FJAT-14178	*Bacillus licheniformis*	nd	2.27±0.02	nd	nd	nd	nd
68	FJAT-14181	*Bacillus pumilus*	nd	2.43±0.01	nd	nd	nd	nd
69	FJAT-14322	*Bacillus pumilus*	nd	1.82±0.06	nd	nd	nd	nd
70	FJAT-14352	*Bacillus pumilus*	nd	2.11±0.33	nd	nd	nd	nd
71	FJAT-14362	*Bacillus pumilus*	nd	2.33±0.18	nd	nd	nd	nd
72	FJAT-14377	*Bacillus licheniformis*	nd	2.7±0.01	nd	nd	nd	nd
73	FJAT-14384	*Bacillus licheniformis*	nd	nd	nd	nd	nd	nd
74	FJAT-14391	*Bacillus licheniformis*	nd	1.22±0.02	nd	nd	nd	nd
75	FJAT-14396	*Bacillus pumilus*	nd	nd	nd	nd	nd	nd
76	FJAT-14491	*Bacillus pumilus*	nd	1.89±0.07	nd	nd	nd	nd
77	FJAT-14528	*Bacillus pumilus*	nd	2.37±0.05	nd	nd	nd	nd
78	FJAT-14647	*Bacillus pumilus*	nd	2.31±0.26	nd	nd	nd	nd
79	FJAT-14704	*Bacillus pumilus*	nd	1.98±0.05	nd	nd	nd	nd
80	FJAT-14725	*Bacillus pumilus*	nd	2.55±0.17	nd	nd	nd	nd
81	FJAT-14726	*Bacillus pumilus*	nd	nd	nd	nd	nd	nd
82	FJAT-14736	*Bacillus pumilus*	nd	nd	nd	nd	nd	nd
83	FJAT-16047	*Bacillus pumilus*	nd	2.24±0.05	nd	14.07±0.19	nd	nd
84	FJAT-16063	*Bacillus pumilus*	nd	nd	nd	nd	nd	nd
85	FJAT-16114	*Bacillus pumilus*	nd	2.71±0.01	nd	nd	nd	nd
86	FJAT-16286	*Bacillus licheniformis*	nd	nd	nd	nd	nd	nd
87	FJAT-16291	*Bacillus pumilus*	nd	nd	nd	nd	nd	nd
88	FJAT-16389	*Bacillus licheniformis*	nd	2.43±0.08	nd	nd	nd	nd
89	FJAT-16398	*Bacillus licheniformis*	nd	1.18±0.04	nd	nd	nd	17.10±0.31
90	FJAT-16414	*Bacillus licheniformis*	2.43±0.31	1.57±0.21	1.54±0.06	nd	nd	nd
91	FJAT-16467	*Bacillus pumilus*	4.02±0.24	1.77±0.13	1.16±0.04	nd	nd	nd
92	FJAT-16564	*Bacillus pumilus*	nd	1.91±0.07	nd	nd	nd	15.96±0.09
93	FJAT-16565	*Bacillus pumilus*	nd	nd	nd	nd	nd	nd
94	FJAT-16578	*Bacillus pumilus*	nd	2.5±0.09	nd	nd	nd	nd
95	FJAT-16611	*Bacillus pumilus*	nd	2.16±0.14	nd	nd	nd	nd
96	FJAT-16705	*Bacillus licheniformis*	2.72±0.21	2.66±0.17	nd	nd	nd	nd
97	FJAT-16766	*Bacillus licheniformis*	1.73±0.01	1.51±0.11	nd	nd	nd	nd
98	FJAT-16777	*Bacillus licheniformis*	2.37±0.2	1.5±0.03	1.25±0.08	nd	nd	nd
99	FJAT-16783	*Bacillus licheniformis*	3.42±0.35	1.64±0.01	1.26±0.04	nd	nd	nd
100	FJAT-16973	*Bacillus pumilus*	nd	nd	nd	nd	nd	nd
101	FJAT-17125	*Bacillus pumilus*	nd	nd	nd	nd	nd	nd
102	FJAT-17127	*Bacillus pumilus*	nd	nd	nd	nd	nd	nd

103	FJAT-17130	*Bacillus pumilus*	nd	nd	nd	nd	nd	nd
104	FJAT-17135	*Bacillus pumilus*	nd	nd	nd	nd	nd	nd
105	FJAT-17137	*Bacillus pumilus*	nd	nd	nd	nd	nd	nd
106	FJAT-17144	*Bacillus pumilus*	nd	nd	nd	nd	nd	nd
107	FJAT-17153	*Bacillus licheniformis*	nd	nd	nd	nd	nd	nd
108	FJAT-17199	*Bacillus licheniformis*	2.46±0.07	1.87±0.05	1.23±0.05	nd	nd	nd
109	FJAT-17228	*Bacillus licheniformis*	2.5±0.45	1.72±0.11	1.4±0.07	nd	nd	nd
110	FJAT-17229	*Bacillus pumilus*	nd	nd	nd	nd	nd	nd
111	FJAT-17230	*Bacillus pumilus*	nd	nd	nd	nd	nd	nd
112	FJAT-17404	*Bacillus pumilus*	nd	nd	nd	nd	nd	nd
113	FJAT-17408	*Bacillus pumilus*	nd	nd	nd	nd	nd	nd
114	FJAT-17419	*Bacillus pumilus*	nd	nd	nd	nd	nd	nd
115	FJAT-17420	*Bacillus pumilus*	nd	nd	nd	nd	nd	nd
116	FJAT-17426	*Bacillus pumilus*	nd	nd	nd	nd	nd	nd
117	FJAT-17429	*Bacillus pumilus*	nd	nd	nd	nd	nd	nd
118	FJAT-17432	*Bacillus licheniformis*	nd	nd	nd	nd	nd	nd
119	FJAT-17433	*Bacillus pumilus*	2.98±0.19	1.26±0.05	1.34±0.01	nd	nd	nd
120	FJAT-17434	*Bacillus pumilus*	nd	nd	nd	nd	nd	nd
121	FJAT-17444	*Bacillus pumilus*	nd	nd	nd	nd	nd	nd
122	FJAT-17451	*Bacillus pumilus*	nd	1.16±0.07	nd	nd	nd	nd
123	FJAT-17457	*Bacillus pumilus*	nd	nd	nd	nd	nd	nd
124	FJAT-17470	*Bacillus pumilus*	nd	nd	nd	nd	nd	nd
125	FJAT-17712	*Bacillus pumilus*	nd	nd	nd	nd	nd	nd
126	FJAT-17726	*Bacillus licheniformis*	nd	nd	nd	nd	nd	nd
127	FJAT-17730	*Bacillus cereus*	2.26±0.2	1.21±0.08	1.24±0.05	nd	16.42±1.03	nd
128	FJAT-17812	*Bacillus licheniformis*	1.8±0.12	1.83±0.08	1.83±0.36	nd	13.87±1.35	nd
129	FJAT-17858	*Bacillus licheniformis*	2.67±0.4	1.25±0.01	1.31±0.01	nd	nd	nd
130	FJAT-18070	*Bacillus licheniformis*	2.59±0.19	1.32±0.05	1.56±0.01	nd	nd	nd
131	FJAT-18098	*Bacillus licheniformis*	2.02±0.08	1.35±0.04	1.16±0.02	nd	nd	nd
132	FJAT-18232	*Bacillus licheniformis*	2.07±0.3	1.53±0.09	1.65±0.17	nd	nd	nd
133	FJAT-18260	*Bacillus licheniformis*	2.48±0.1	1.67±0.25	1.57±0.01	nd	nd	nd
134	FJAT-18269	*Bacillus pumilus*	2.23±0.21	1.43±0.13	1.52±0.15	nd	nd	nd
135	FJAT-18285	*Bacillus licheniformis*	nd	1.91±0.13	nd	nd	nd	nd
136	FJAT-18287	*Bacillus pumilus*	1.99±0.01	1.35±0.09	1.52±0.08	nd	nd	nd
137	FJAT-18490	*Bacillus pumilus*	nd	1.79±0.22	nd	nd	nd	nd
138	FJAT-18516	*Bacillus pumilus*	nd	nd	nd	nd	nd	nd
139	FJAT-18542	*Bacillus licheniformis*	nd	1.89±0.26	nd	18.46±0.27	nd	nd
140	FJAT-18549	*Bacillus licheniformis*	1.63±0.02	1.3±0.12	1.82±0.18	nd	nd	nd
141	FJAT-18565	*Bacillus pumilus*	1.47±0.12	1.44±0.15	1.77±0.04	nd	nd	nd
142	FJAT-18587	*Bacillus licheniformis*	nd	1.82±0.16	nd	nd	nd	nd
143	FJAT-18590	*Bacillus pumilus*	3.77±0.48	1.83±0.17	1.44±0.01	nd	nd	nd
144	FJAT-18619	*Bacillus pumilus*	nd	2.44±0.17	nd	nd	nd	nd
145	FJAT-18900	*Bacillus pumilus*	nd	1.97±0.14	nd	nd	nd	nd
146	FJAT-18901	*Bacillus pumilus*	nd	1.86±0.16	nd	nd	nd	nd
147	FJAT-18903	*Bacillus pumilus*	nd	1.75±0.01	nd	nd	nd	nd
148	FJAT-18906	*Bacillus pumilus*	nd	1.89±0.16	nd	19.84±0.17	nd	nd
149	FJAT-18909	*Bacillus pumilus*	nd	1.91±0.08	nd	nd	nd	nd
150	FJAT-18912	*Bacillus pumilus*	nd	nd	nd	nd	nd	nd
151	FJAT-18916	*Bacillus pumilus*	nd	2.08±0.18	nd	nd	nd	nd
152	FJAT-18920	*Bacillus pumilus*	nd	1.81±0.18	nd	nd	nd	nd

153	FJAT-18921	*Bacillus pumilus*	nd	1.97±0.11	nd	nd	nd	nd
154	FJAT-18925	*Bacillus pumilus*	nd	1.73±0.17	nd	nd	nd	nd
155	FJAT-18926	*Bacillus pumilus*	nd	2.8±0.23	nd	nd	nd	nd
156	FJAT-18927	*Bacillus pumilus*	nd	1.72±0.01	nd	nd	nd	nd
157	FJAT-18930	*Bacillus pumilus*	nd	1.74±0.13	nd	nd	nd	nd
158	FJAT-18934	*Bacillus pumilus*	nd	1.79±0.32	nd	nd	nd	nd
159	FJAT-18937	*Bacillus licheniformis*	nd	2.09±0.15	nd	nd	nd	nd
160	FJAT-18945	*Bacillus pumilus*	3.1±0.16	1.18±0.05	1.25±0.01	nd	nd	nd
161	FJAT-18946	*Bacillus pumilus*	nd	1.87±0.29	nd	nd	nd	nd
162	FJAT-18955	*Bacillus pumilus*	nd	2.1±0.16	nd	nd	nd	nd
163	FJAT-18958	*Bacillus pumilus*	nd	2.09±0.03	nd	nd	nd	nd
164	FJAT-18959	*Bacillus pumilus*	nd	3.5±0.32	nd	nd	nd	nd
165	FJAT-18961	*Bacillus pumilus*	nd	2.74±0.24	nd	14.23±0.11	nd	nd
166	FJAT-18962	*Bacillus pumilus*	nd	1.92±0.15	nd	nd	nd	nd
167	FJAT-18963	*Bacillus pumilus*	nd	2.12±0.04	nd	nd	nd	nd
168	FJAT-18964	*Bacillus pumilus*	nd	1.94±0.01	nd	nd	nd	nd
169	FJAT-18968	*Bacillus pumilus*	nd	2±0.02	nd	nd	nd	nd
170	FJAT-18970	*Bacillus licheniformis*	nd	2.54±0.07	nd	nd	nd	nd
171	FJAT-18973	*Bacillus licheniformis*	2.33±0.67	1.49±0.17	1.27±0.07	nd	nd	nd
172	FJAT-19876	*Bacillus pumilus*	nd	1.66±0.37	nd	nd	nd	nd
173	FJAT-19890	*Bacillus licheniformis*	nd	2.6±0.44	nd	nd	nd	nd
174	FJAT-20275	*Bacillus pumilus*	1.89±0.05	1.28±0.04	1.55±0.16	nd	nd	nd
175	FJAT-20278	*Bacillus pumilus*	nd	2.05±0.15	nd	nd	nd	nd
176	FJAT-20313	*Bacillus pumilus*	nd	1.77±0.08	nd	nd	nd	nd
177	FJAT-20317	*Bacillus pumilus*	nd	1.97±0.25	nd	nd	nd	nd
178	FJAT-20328	*Bacillus pumilus*	nd	2.05±0.17	nd	nd	nd	nd
179	FJAT-20329	*Bacillus pumilus*	nd	2.04±0.02	nd	17.52±0.29	nd	nd
180	FJAT-20636	*Bacillus licheniformis*	nd	2.05±0.09	nd	nd	nd	nd
181	FJAT-20674	*Bacillus pumilus*	2.19±0.16	1.43±0.04	nd	nd	nd	nd
182	FJAT-21361	*Bacillus pumilus*	nd	2.1±0.05	nd	18.22±0.31	nd	nd
183	FJAT-21365	*Bacillus pumilus*	nd	1.84±0.16	nd	nd	nd	nd
184	FJAT-21381	*Bacillus pumilus*	nd	1.9±0.21	nd	nd	nd	nd
185	FJAT-21391	*Bacillus pumilus*	nd	1.82±0.21	nd	nd	nd	nd
186	FJAT-21426	*Bacillus pumilus*	nd	1.79±0.13	nd	nd	nd	nd
187	FJAT-21888	*Bacillus pumilus*	nd	1.86±0.16	nd	13.11±0.15	nd	nd
188	FJAT-21898	*Bacillus pumilus*	nd	2.26±0.07	nd	nd	nd	nd
189	FJAT-21899	*Bacillus pumilus*	nd	2.41±0.01	nd	14.32±0.14	nd	nd
190	FJAT-21900	*Bacillus pumilus*	nd	2.2±0.02	nd	13.97±0.32	nd	nd
191	FJAT-21936	*Bacillus pumilus*	nd	2.23±0.13	nd	nd	nd	nd
192	FJAT-21937	*Bacillus pumilus*	nd	2.1±0.06	nd	nd	nd	nd
193	FJAT-21943	*Bacillus pumilus*	nd	2.42±0.24	nd	nd	nd	nd
194	FJAT-21946	*Bacillus pumilus*	nd	2.17±0.03	nd	nd	nd	nd
195	FJAT-21947	*Bacillus pumilus*	nd	2.21±0.24	nd	nd	nd	nd
196	FJAT-21949	*Bacillus pumilus*	nd	nd	nd	nd	nd	nd
197	FJAT-21952	*Bacillus pumilus*	nd	2.69±0.1	nd	nd	nd	nd
198	FJAT-21959	*Bacillus pumilus*	nd	2.11±0.1	nd	nd	nd	nd
199	FJAT-21968	*Bacillus pumilus*	nd	1.94±0.15	nd	nd	nd	nd
200	FJAT-21975	*Bacillus pumilus*	nd	2.43±0.48	nd	nd	nd	nd
201	FJAT-21987	*Bacillus pumilus*	nd	1.76±0.02	nd	nd	nd	nd
202	FJAT-21989	*Bacillus pumilus*	nd	1.84±0.05	nd	nd	nd	nd

203	FJAT-21990	*Bacillus pumilus*	nd	2.29±0.43	nd	nd	nd	nd
204	FJAT-22059	*Bacillus pumilus*	nd	2.32±0.25	nd	nd	nd	nd
205	FJAT-22069	*Bacillus pumilus*	nd	1.9±0.1	nd	nd	nd	nd
206	FJAT-22071	*Bacillus licheniformis*	nd	1.93±0.14	nd	nd	nd	nd
207	FJAT-22085	*Bacillus pumilus*	2.69±1.02	1.4±0.11	nd	nd	nd	14.72±0.25
208	FJAT-22102	*Bacillus pumilus*	nd	2.05±0.27	nd	nd	nd	nd
209	FJAT-22117	*Bacillus pumilus*	nd	2.04±0.35	nd	nd	nd	nd
210	FJAT-22135	*Bacillus pumilus*	nd	2.08±0.13	nd	nd	nd	nd
211	FJAT-22177	*Bacillus pumilus*	nd	nd	nd	nd	nd	nd
212	FJAT-22402	*Bacillus pumilus*	nd	2.21±0.47	nd	nd	nd	nd
213	FJAT-22404	*Bacillus pumilus*	nd	1.89±0.39	nd	nd	nd	nd
214	FJAT-22409	*Bacillus pumilus*	nd	1.57±0.11	nd	nd	nd	nd
215	FJAT-22411	*Bacillus pumilus*	nd	1.76±0.19	nd	nd	nd	nd
216	FJAT-22413	*Bacillus pumilus*	nd	1.8±0.22	nd	nd	nd	nd
217	FJAT-22419	*Bacillus licheniformis*	nd	2.59±0.25	nd	nd	nd	nd
218	FJAT-22421	*Bacillus pumilus*	2±0.08	1.58±0.01	1.19±0.05	nd	nd	nd
219	FJAT-22423	*Bacillus pumilus*	nd	2.39±0.14	nd	nd	nd	nd
220	FJAT-22424	*Bacillus pumilus*	nd	1.7±0.01	nd	nd	nd	nd
221	FJAT-22425	*Bacillus licheniformis*	nd	2±0.04	nd	nd	nd	nd
222	FJAT-22448	*Bacillus licheniformis*	3.16±0.36	1.57±0.17	1.28±0.1	nd	nd	nd
223	FJAT-23288	*Bacillus licheniformis*	4.26±0.84	1.6±0.3	1.3±0.17	nd	nd	nd
224	FJAT-23289	*Bacillus licheniformis*	2.97±0.01	1.52±0.19	1.32±0.03	nd	nd	nd
225	FJAT-23292	*Bacillus licheniformis*	2.37±0.44	2.17±0.32	1.47±0.01	nd	nd	nd
226	FJAT-23456	*Bacillus licheniformis*	2.21±0.17	nd	1.43±0.04	nd	nd	nd
227	FJAT-23552	*Bacillus licheniformis*	1.81±0.08	nd	nd	nd	nd	nd
228	FJAT-23554	*Bacillus licheniformis*	2.35±0.13	nd	nd	nd	nd	nd
229	FJAT-23598	*Bacillus licheniformis*	2.38±0.42	1.71±0.09	1.47±0.11	nd	nd	nd
230	FJAT-25445	*Bacillus licheniformis*	nd	1.55±0.01	nd	nd	nd	nd
231	FJAT-25449	*Bacillus licheniformis*	2.62±0.35	1.91±0.21	1.54±0.01	nd	nd	nd
232	FJAT-25450	*Bacillus licheniformis*	1.98±0.16	1.62±0.36	1.46±0.1	nd	12.32±0.04	nd
233	FJAT-25451	*Bacillus licheniformis*	nd	1.33±0.08	nd	nd	nd	nd
234	FJAT-25463	*Bacillus licheniformis*	2.2±0.43	1.84±0.05	1.71±0.08	nd	nd	nd
235	FJAT-25682	*Bacillus licheniformis*	1.93±0.44	1.07±0.02	nd	nd	nd	nd
236	FJAT-25742	*Bacillus licheniformis*	1.79±0.28	1.76±0.01	1.57±0.12	nd	nd	nd
237	FJAT-25795	*Bacillus licheniformis*	2.19±0.23	nd	1.39±0.15	nd	nd	nd
238	FJAT-25889	*Bacillus licheniformis*	1.83±0.38	nd	1.79±0.01	nd	nd	nd
239	FJAT-25895	*Bacillus licheniformis*	1.83±0.26	1.29±0.05	1.21±0.14	nd	nd	nd
240	FJAT-26038	*Bacillus licheniformis*	2.04±0.1	1.16±0.03	nd	11.35±0.12	nd	nd
241	FJAT-26039	*Bacillus licheniformis*	2.26±0.29	1.47±0.36	2.04±0.1	nd	nd	nd
242	FJAT-26043	*Bacillus licheniformis*	2.66±0.31	1.98±0.14	1.45±0.11	nd	nd	nd
243	FJAT-26143	*Bacillus licheniformis*	1.89±0.11	1.3±0.03	1.59±0.01	nd	nd	nd
244	FJAT-26145	*Bacillus licheniformis*	1.55±0.06	1.22±0.01	1.28±0.08	nd	nd	nd
245	FJAT-26148	*Bacillus licheniformis*	nd	nd	nd	nd	nd	nd
246	FJAT-26149	*Bacillus licheniformis*	2.08±0.07	1.69±0.45	1.97±0.01	nd	nd	nd
247	FJAT-26150	*Bacillus licheniformis*	3.43±0.38	1.64±0.1	1.49±0.01	nd	nd	nd
248	FJAT-26163	*Bacillus licheniformis*	2.01±0.03	nd	nd	nd	nd	nd
249	FJAT-26303	*Bacillus licheniformis*	2.19±0.19	1.75±0.41	nd	nd	nd	nd
250	FJAT-26305	*Bacillus pumilus*	2.89±0.12	1.1±0.01	1.54±0.15	nd	nd	nd
251	FJAT-26306	*Bacillus licheniformis*	1.33±0.07	1.95±0.08	1.39±0.22	nd	15.08±1.09	nd
252	FJAT-26312	*Bacillus licheniformis*	2.05±0.12	nd	2.08±0.09	nd	nd	nd

253	FJAT-26319	*Bacillus licheniformis*	2.11±0.29	1.28±0.12	1.61±0.13	nd	nd	nd
254	FJAT-26320	*Bacillus licheniformis*	2.02±0.12	1.73±0.09	1.72±0.2	nd	nd	nd
255	FJAT-26325	*Bacillus licheniformis*	2.44±0.4	1.34±0.04	1.33±0.07	nd	nd	nd
256	FJAT-26329	*Bacillus licheniformis*	1.62±0.07	1.12±0.03	1.36±0.01	nd	nd	nd
257	FJAT-26330	*Bacillus licheniformis*	3.24±0.75	1.36±0.14	1.9±0.11	nd	nd	nd
258	FJAT-26403	*Bacillus licheniformis*	2.3±0.21	1.36±0.06	1.58±0.14	nd	nd	nd
259	FJAT-26486	*Bacillus licheniformis*	1.76±0.03	nd	1.45±0.2	nd	nd	nd
260	FJAT-26495	*Bacillus licheniformis*	1.99±0.22	2.05±0.28	1.4±0.02	nd	nd	nd
261	FJAT-26610	*Bacillus licheniformis*	2.79±0.53	1.34±0.03	1.5±0.01	nd	nd	nd
262	FJAT-26686	*Bacillus pumilus*	nd	1.58±0.14	nd	nd	nd	nd
263	FJAT-26739	*Bacillus licheniformis*	1.56±0.11	1.14±0.06	1.24±0.1	nd	nd	nd
264	FJAT-26744	*Bacillus licheniformis*	nd	nd	nd	nd	nd	nd
265	FJAT-26745	*Bacillus licheniformis*	1.8±0.3	1.47±0.32	1.58±0.15	nd	nd	nd
266	FJAT-26747	*Bacillus licheniformis*	2.54±0.65	1.57±0.16	1.75±0.1	nd	nd	nd
267	FJAT-26748	*Bacillus licheniformis*	3.05±0.65	1.89±0.34	1.72±0.21	nd	nd	nd
268	FJAT-27193	*Bacillus licheniformis*	2.84±0.21	1.41±0.15	1.12±0.08	nd	nd	nd
269	FJAT-27197	*Bacillus licheniformis*	1.81±0.06	1.24±0.02	nd	nd	nd	nd
270	FJAT-27199	*Bacillus licheniformis*	1.54±0.13	1.4±0.03	1.49±0.17	nd	nd	nd
271	FJAT-27200	*Bacillus licheniformis*	1.95±0.01	1.29±0.12	1.8±0.07	nd	nd	nd
272	FJAT-27213	*Bacillus licheniformis*	2.93±0.14	1.26±0.02	1.49±0.03	nd	nd	nd
273	FJAT-27214	*Bacillus licheniformis*	1.51±0.03	1.23±0.08	1.33±0.09	nd	nd	nd
274	FJAT-27220	*Bacillus licheniformis*	2.65±1.14	1.51±0.01	nd	nd	nd	nd
275	FJAT-27240	*Bacillus licheniformis*	1.7±0.2	1.27±0.05	1.44±0.22	nd	nd	nd
276	FJAT-27642	*Bacillus licheniformis*	2.12±0.26	nd	1.37±0.03	nd	nd	nd
277	FJAT-27644	*Bacillus licheniformis*	1.91±0.14	nd	nd	nd	nd	14.49±0.17
278	FJAT-27675	*Bacillus licheniformis*	3.7±0.09	nd	1.47±0.14	nd	nd	nd
279	FJAT-27678	*Bacillus licheniformis*	2.13±0.35	1.32±0.08	1.48±0.08	nd	nd	nd
280	FJAT-27704	*Bacillus licheniformis*	1.84±0.32	1.44±0.01	nd	nd	nd	nd
281	FJAT-27705	*Bacillus licheniformis*	2.44±0.98	2.19±0.19	nd	nd	nd	nd
282	FJAT-27709	*Bacillus licheniformis*	2.13±0.42	1.65±0.11	1.67±0.03	nd	nd	nd
283	FJAT-27712	*Bacillus licheniformis*	1.84±0.07	1.75±0.11	nd	nd	nd	nd
284	FJAT-27713	*Bacillus licheniformis*	nd	nd	nd	nd	nd	nd
285	FJAT-27722	*Bacillus licheniformis*	1.74±0.07	1.15±0.05	1.22±0.05	nd	nd	nd
286	FJAT-27724	*Bacillus licheniformis*	2.25±0.89	1.54±0.23	nd	nd	nd	nd
287	FJAT-27725	*Bacillus licheniformis*	2.24±0.32	1.32±0.07	nd	nd	nd	nd
288	FJAT-27726	*Bacillus licheniformis*	1.86±0.64	2.19±0.61	nd	nd	nd	nd
289	FJAT-27730	*Bacillus licheniformis*	2.07±0.31	1.4±0.13	1.28±0.05	nd	nd	nd
290	FJAT-29686	*Bacillus licheniformis*	nd	2.3±0.44	nd	nd	nd	nd
291	FJAT-29687	*Bacillus licheniformis*	nd	2.61±0.72	nd	nd	nd	nd
292	FJAT-29849	*Bacillus licheniformis*	nd	1.29±0.04	nd	nd	nd	nd
293	FJAT-29855	*Bacillus licheniformis*	3.12±0.55	1.3±0.2	1.53±0.02	nd	nd	nd
294	FJAT-40180	*Bacillus licheniformis*	2.13±0.01	1.55±0.04	nd	nd	nd	nd
295	FJAT-40181	*Bacillus licheniformis*	2.15±0.65	1.66±0.32	1.5±0.18	nd	nd	nd
296	FJAT-40190	*Bacillus licheniformis*	2.48±0.84	1.66±0.15	1.82±0.01	nd	nd	nd
297	FJAT-40194	*Bacillus licheniformis*	2.63±0.18	1.87±0.06	nd	nd	nd	nd
298	FJAT-40205	*Bacillus licheniformis*	2.17±0.24	1.35±0.16	nd	nd	nd	nd
299	FJAT-40215	*Bacillus licheniformis*	2.23±0.21	1.49±0.04	1.35±0.04	nd	nd	nd
300	FJAT-40232	*Bacillus licheniformis*	2.55±0.14	1.63±0.08	1.9±0.25	nd	14.93±0.11	nd
301	FJAT-40250	*Bacillus licheniformis*	2.69±0.21	1.42±0.03	nd	nd	nd	nd
302	FJAT-40253	*Bacillus licheniformis*	2.27±0.66	1.45±0.01	nd	nd	nd	nd

303	FJAT-40254	*Bacillus licheniformis*	2.31±0.01	nd	nd	nd	nd	nd
304	FJAT-40256	*Bacillus licheniformis*	2.48±0.07	1.18±0.04	1.42±0.1	nd	nd	nd
305	FJAT-40258	*Bacillus licheniformis*	2.69±0.18	1.49±0.09	1.21±0.02	nd	nd	nd
306	FJAT-40267	*Bacillus licheniformis*	2.43±0.56	1.16±0.07	1.51±0.13	nd	nd	nd
307	FJAT-40269	*Bacillus licheniformis*	2.21±0.12	nd	nd	nd	nd	20.28±0.28
308	FJAT-40272	*Bacillus licheniformis*	2.15±0.28	nd	1.44±0.27	nd	nd	nd
309	FJAT-40273	*Bacillus licheniformis*	1.82±0.24	nd	nd	nd	nd	nd
310	FJAT-41160	*Bacillus licheniformis*	2.55±0.26	1.44±0.07	1.95±0.04	nd	nd	nd
311	FJAT-41168	*Bacillus licheniformis*	1.99±0.08	1.53±0.01	1.2±0.04	nd	nd	nd
312	FJAT-41173	*Bacillus licheniformis*	1.95±0.17	1.21±0.14	nd	nd	nd	nd
313	FJAT-41177	*Bacillus licheniformis*	1.73±0.21	nd	nd	nd	nd	nd
314	FJAT-41184	*Bacillus licheniformis*	1.72±0.1	1.22±0.06	1.76±0.11	nd	nd	nd
315	FJAT-41191	*Bacillus licheniformis*	1.65±0.03	1.59±0.21	1.39±0.03	nd	nd	nd
316	FJAT-41204	*Bacillus licheniformis*	2.19±0.07	nd	1.52±0.02	nd	nd	nd
317	FJAT-41235	*Bacillus licheniformis*	1.86±0.02	1.4±0.19	1.53±0.11	nd	nd	nd
318	FJAT-41239	*Bacillus licheniformis*	2.16±0.53	1.4±0.02	nd	nd	nd	nd
319	FJAT-41240	*Bacillus licheniformis*	1.92±0.01	1.27±0.05	1.56±0.21	nd	nd	nd
320	FJAT-41267	*Bacillus licheniformis*	nd	nd	nd	nd	nd	nd
321	FJAT-41269	*Bacillus licheniformis*	nd	nd	nd	nd	nd	nd
322	FJAT-41272	*Bacillus licheniformis*	2.91±0.3	1.16±0.05	nd	nd	nd	nd
323	FJAT-41275	*Bacillus licheniformis*	2.04±0.02	1.42±0.08	1.4±0.04	nd	nd	nd
324	FJAT-41277	*Bacillus licheniformis*	1.91±0.09	1.79±0.1	nd	nd	nd	nd
325	FJAT-41289	*Bacillus licheniformis*	2.18±0.35	1.33±0.1	1.16±0.09	nd	nd	nd
326	FJAT-41293	*Bacillus licheniformis*	2.23±0.15	nd	nd	nd	nd	nd
327	FJAT-41294	*Bacillus licheniformis*	2.07±0.01	nd	nd	nd	16.50±0.16	nd
328	FJAT-41297	*Bacillus licheniformis*	1.45±0.12	1.37±0.04	1.21±0.07	nd	nd	nd
329	FJAT-41298	*Bacillus licheniformis*	1.9±0.04	1.27±0.03	1.54±0.09	nd	nd	nd
330	FJAT-41302	*Bacillus licheniformis*	1.71±0.23	1.62±0.12	nd	nd	nd	nd
331	FJAT-41304	*Bacillus licheniformis*	2.67±0.16	1.3±0.03	1.2±0.04	nd	nd	nd
332	FJAT-41324	*Bacillus licheniformis*	2.18±0.13	1.58±0.09	nd	nd	nd	21.18±0.35
333	FJAT-41325	*Bacillus licheniformis*	nd	nd	nd	nd	nd	nd
334	FJAT-41332	*Bacillus licheniformis*	1.49±0.03	1.41±0.12	1.64±0.01	nd	nd	nd
335	FJAT-41341	*Bacillus licheniformis*	1.64±0.06	nd	1.44±0.06	nd	nd	nd
336	FJAT-41343	*Bacillus licheniformis*	2.46±0.19	2.14±0.54	1.45±0.14	nd	nd	nd
337	FJAT-41600	*Bacillus licheniformis*	2.23±0.23	1.36±0.07	nd	nd	nd	nd
338	FJAT-41609	*Bacillus licheniformis*	4.04±0.24	nd	nd	nd	nd	nd
339	FJAT-41613	*Bacillus licheniformis*	2.16±0.38	nd	nd	nd	nd	nd
340	FJAT-41615	*Bacillus licheniformis*	2.27±0.58	1.39±0.1	nd	nd	nd	nd
341	FJAT-41630	*Bacillus licheniformis*	1.86±0.42	1.75±0.26	nd	nd	nd	nd
342	FJAT-41643	*Bacillus licheniformis*	1.75±0.3	2.05±0.11	nd	nd	nd	nd
343	FJAT-41654	*Bacillus licheniformis*	1.96±0.01	1.73±0.21	1.2±0.03	nd	nd	nd
344	FJAT-41670	*Bacillus licheniformis*	1.81±0.01	1.33±0.02	1.48±0.05	nd	nd	nd
345	FJAT-41685	*Bacillus licheniformis*	2.88±0.05	3.04±0.77	1.36±0.09	nd	nd	nd
346	FJAT-41686	*Bacillus licheniformis*	1.87±0.41	1.4±0.03	nd	nd	nd	nd
347	FJAT-41687	*Bacillus licheniformis*	2.5±0.34	nd	nd	nd	nd	nd
348	FJAT-41706	*Bacillus licheniformis*	3.49±1.26	nd	1.47±0.13	nd	nd	nd
349	FJAT-41727	*Bacillus licheniformis*	1.99±0.12	1.59±0.02	1.48±0.03	nd	nd	nd
350	FJAT-41730	*Bacillus licheniformis*	2.01±0.52	2.04±0.07	1.32±0.07	nd	nd	nd
351	FJAT-41737	*Bacillus licheniformis*	1.65±0.21	nd	nd	nd	nd	nd
352	FJAT-41738	*Bacillus licheniformis*	2.36±0.23	1.4±0.04	1.47±0.09	nd	nd	nd

353	FJAT-41741	*Bacillus licheniformis*	2.51±0.33	nd	1.5±0.04	nd	nd	nd
354	FJAT-41744	*Bacillus licheniformis*	2.51±0.29	1.37±0.08	1.28±0.02	nd	nd	nd
355	FJAT-41745	*Bacillus licheniformis*	nd	1.5±0.03	1.45±0.09	nd	nd	nd
356	FJAT-41749	*Bacillus licheniformis*	1.81±0.05	1.27±0.04	1.65±0.08	nd	nd	nd
357	FJAT-41762	*Bacillus licheniformis*	3.44±0.18	1.53±0.07	1.94±0.04	nd	nd	nd
358	FJAT-41763	*Bacillus licheniformis*	2.83±0.32	1.67±0.3	1.72±0.01	nd	nd	nd
359	FJAT-41766	*Bacillus licheniformis*	2.84±0.8	nd	nd	nd	nd	nd
360	FJAT-41767	*Bacillus licheniformis*	2.79±0.23	nd	1.8±0.12	nd	nd	nd
361	FJAT-41768	*Bacillus licheniformis*	1.6±0.03	1.09±0.04	1.36±0.05	nd	nd	nd
362	FJAT-41770	*Bacillus licheniformis*	2.62±0.75	1.28±0.12	1.49±0.01	nd	nd	nd
363	FJAT-44199	*Bacillus licheniformis*	3.25±0.61	1.16±0.07	1.8±0.11	nd	nd	nd
364	FJAT-44200	*Bacillus licheniformis*	2.73±0.02	1.45±0.34	1.63±0.17	nd	nd	nd
365	FJAT-44204	*Bacillus licheniformis*	2.19±0.01	1.36±0.19	1.58±0.14	nd	nd	nd
366	FJAT-44205	*Bacillus licheniformis*	2.76±0.15	1.3±0.16	1.51±0.01	nd	nd	nd
367	FJAT-44505	*Bacillus licheniformis*	3.53±0.1	1.18±0.02	1.28±0.01	nd	nd	nd
368	FJAT-44545	*Bacillus licheniformis*	1.99±0.35	1.37±0.21	1.26±0.06	nd	nd	nd
369	FJAT-44552	*Bacillus licheniformis*	1.96±0.28	1.42±0.14	1.37±0.06	nd	18.77±0.21	nd
370	FJAT-44648	*Bacillus licheniformis*	2.2±0.03	2.19±0.02	2.02±0.51	nd	nd	nd
371	FJAT-44662	*Bacillus licheniformis*	2.64±0.18	1.8±0.09	1.38±0.14	nd	nd	nd
372	FJAT-44682	*Bacillus licheniformis*	2.74±0.19	1.46±0.04	1.59±0.03	nd	nd	nd
373	FJAT-44684	*Bacillus licheniformis*	2.95±0.94	1.63±0.05	1.47±0.02	nd	nd	nd
374	FJAT-44695	*Bacillus licheniformis*	4.19±0.84	nd	nd	nd	nd	nd
375	FJAT-44697	*Bacillus licheniformis*	nd	1.84±0.01	1.5±0.06	nd	nd	nd
376	FJAT-44717	*Bacillus licheniformis*	nd	nd	nd	nd	nd	nd
377	FJAT-44745	*Bacillus pumilus*	2.65±0.34	1.5±0.11	1.44±0.03	nd	nd	nd
378	FJAT-44833	*Bacillus licheniformis*	2.87±0.57	1.55±0.22	1.51±0.23	nd	nd	nd
379	FJAT-44891	*Bacillus licheniformis*	2.77±0.54	1.59±0.1	1.54±0.01	nd	nd	nd
380	FJAT-44931	*Bacillus licheniformis*	2.58±0.18	1.54±0.03	2.18±0.19	nd	nd	nd
381	FJAT-45099	*Bacillus licheniformis*	3.13±0.16	1.33±0.02	1.43±0.02	nd	nd	nd
382	FJAT-45139	*Bacillus licheniformis*	1.85±0.25	1.42±0.06	1.28±0.05	nd	nd	nd
383	FJAT-45140	*Bacillus licheniformis*	nd	3.42±0.12	nd	nd	nd	nd
384	FJAT-45249	*Bacillus licheniformis*	3.29±0.88	1.45±0.14	1.44±0.11	nd	nd	nd
385	FJAT-45294	*Bacillus licheniformis*	nd	nd	nd	nd	nd	nd
386	FJAT-45364	*Bacillus licheniformis*	3.18±0.39	1.47±0.01	1.72±0.26	nd	nd	nd
387	FJAT-45494	*Bacillus licheniformis*	1.91±0.12	1.71±0.25	1.41±0.09	nd	nd	nd
388	FJAT-45541	*Bacillus licheniformis*	2.73±0.37	1.71±0.06	1.79±0.16	nd	nd	nd
389	FJAT-46469	*Bacillus licheniformis*	2.74±0.38	1.5±0.01	1.81±0.14	nd	nd	nd
390	FJAT-46567	*Bacillus licheniformis*	2.87±0.05	1.89±0.53	1.83±0.19	nd	nd	nd
391	FJAT-46609	*Bacillus licheniformis*	2.66±0.29	1.79±0.11	1.45±0.04	nd	nd	nd
392	FJAT-46910	*Bacillus licheniformis*	2.31±0.16	1.64±0.01	1.61±0.16	nd	nd	nd
393	FJAT-47580	*Bacillus licheniformis*	3.17±0.33	1.64±0.08	1.75±0.01	nd	nd	nd
394	FJAT-47753	*Bacillus licheniformis*	2.68±0.19	nd	1.63±0.05	nd	nd	nd

Note: # The strain number (FJAT-) was authorized by the Fujian Bacilli Resource Collection Center (FBRCC), Institute of Resources, Environment and Soil Fertilizer, Fujian Academy of Agricultural Sciences. * The taxonomic information of each strain had been validated through 16S rRNA and *gyrB* sequence analyses by FBRCC. Cellulase, protease and amylase activities are expressed as D/d, where D is the diameter of the clearance zone and d is the colony diameter. Antibacterial activity is recorded as inhibition zone diameter (mm). "nd" indicates the absence of a detectable clearance or inhibition zone.

Table S2. Putative virulence genes predicted in the FJAT-10508 and FJAT-13563 genomes

NO.	Functioin description	FJAT-10508	FJAT-13563
1	Adherence	*ilpA, cwpV, fbpA, gbpA, groEL, lap, lmb, pebA, pilD, pilS, rpoN, scpA/scpB, tufA* (13)	*ilpA, cwpV, ecbA/fss3, fbpA, gbpA, groEL, lap, lmb, pebA, pilD, pilS, rpoN, scpA/scpB, tufA* (14)
2	Biofilm	*algC, algD, algI, algR, bopD, icaB, icaC, luxS, mucD, mucP, vpsG, vpsU* (12)	*algC, algD, algI, algR, algU, bopD, icaB, icaC, luxS, mucD, mucP, vpsG, vpsU* (13)
3	Effector delivery system	*CBU_1566, CBU_1789, CT_061, CT_473, LPG_RS14840, PA2359, cdsN, eccA3, esaG, essC, lirB, mycP1, ppkA, pppA, ssrB, tagT, vscN, xcpR, yycJ* (19)	*CBU_1566, CBU_1789, CT_061, CT_473, LPG_RS14840, PA2359, cdsN, eccA3, esaG, essC, lirB, mycP1, ppkA, pppA, tagT, vscN, xcpR, yycJ* (18)
4	Exoenzyme	*aur, cwp84, tlyC* (3)	*aur, cwp84, tlyC* (3)
5	Exotoxin	*cer, cesA, cesB, cesC, cesP, clbB, clbD, clbF, clbG, clbJ, clbK, clbP, clbQ, cyaB, cylA, cylB, cylG, hlyB, llsG, rtxB, rtxE* (21)	*cesA, cesB, cesC, cesP, clbB, clbD, clbF, clbG, clbI, clbJ, clbK, clbP, clbQ, cyaB, cylA, cylB, cylG, cylR2, hlyB, llsG, rtxB, rtxE* (22)
6	Immune modulation	*ACICU_RS00400, ACICU_RS00475, Cj1136, Cj1437c, FTT_RS04105, GBS_RS06565, GBS_RS06570, GBS_RS06600, GBS_RS06610, KP1_RS17220, LPG_RS03745, LPG_RS03830, acpXL, adsA, bplF, bplI, cap8B, cap8C, cap8D, cap8E, capA, capB, capC, cps4H, cps4I, cpsA/uppS, cpsB/cdsA, cpsC, cpsG, cpsJ, ctrD, cysC, ddrA, fabZ, galE, gndA, gtrB, hasC, hisH2, hldD, hldE, kdtB, kfiC, legI, lgtA, lgtC, lgtF, lpxA/glmU, lsgC, lsgE, manB/yhxB, mmpL8, msbA, napA, oatA, orfM, pbpG, pdgA, pks1, pks2, ppsA, ppsB, rfbA, rfbB, rfbD, rfbF, rpe, wbpA, wbpL, wbtD, wbtH, wbtM, wbuZ, wcbR, wcbT, wzt* (76)	*ACICU_RS00400, ACICU_RS00475, Cj1136, Cj1437c, FTT_RS04105, GBS_RS06565, GBS_RS06570, GBS_RS06600, GBS_RS06610, KP1_RS17220, LPG_RS03745, LPG_RS03830, Rv2952, acpXL, adsA, bplF, bplI, cap8B, cap8C, cap8D, cap8E, capA, capB, capC, cps4H, cps4I, cpsA/uppS, cpsB/cdsA, cpsC, cpsG, cpsJ, ctrD, cysC, ddrA, fabZ, galE, gndA, gtrB, hasC, hisH2, hldD, hldE, kdtB, kfiC, legI, lgtA, lgtC, lgtF, lpxA/glmU, lsgC, lsgE, manB/yhxB, mmpL8, msbA, napA, oatA, orfM, pbpG, pdgA, pks1, pks2, ppsA, ppsB, rfbA, rfbB, rfbD, rfbF, rpe, wbpA, wbpL, wbtD, wbtH, wbtM, wbuZ, wcbR, wcbT, wzt* (77)
7	Invasion	*aut, iap/cwhA* (2)	*aut, iap/cwhA* (2)
8	Motility	*AHML_RS07540, Cj0883c, PA3348, cheA,*	*AHML_RS07540, Cj0883c, PA3348, cheA,*

		cheB, cheB-2, cheV, cheV3, cheW, flaA, fleN, fleQ, fleR/flrC, fleS/flrB, flgB, flgC, flgD, flgG2, flgG_2, flgK, flgL, flhA, flhB, flhF, fliD, fliE, fliF, fliG, fliI, fliM, fliN, fliP, fliQ, fliR, fliS, flmH, flrB, lafK, lfgG, motA, motB, motC, pseB, tlpB, tlpC (45)	*cheB, cheB-2, cheV, cheV3, cheW, flaA, fleN, fleQ, fleR/flrC, fleS/flrB, flgB, flgC, flgD, flgG2, flgG_2, flgJ, flgK, flgL, flhA, flhB, flhF, fliD, fliE, fliF, fliG, fliI, fliM, fliN, fliP, fliQ, fliR, fliS, flmH, flrB, lafK, lfgG, motA, motB, motC, pseB, tlpB, tlpC* (46)
9	Others	*aatC* (1)	*aatC* (1)
10	Post-translational modification	*gtcA, lspA, prsA2* (3)	*gtcA, lspA, prsA2* (3)
11	Regulation	*AAD32423, bvgA, bvrR, bvrS, csrA, devR/dosR, devS, letA, letS, mprA, mprB, phoP, phoR, relA, rpoS, sigA/rpoV, sigE, sigF, sigH* (19)	*AAD32423, bvgA, bvgS, bvrR, bvrS, csrA, devR/dosR, devS, letA, letS, mprA, mprB, phoP, phoR, relA, rpoS, sigA/rpoV, sigE, sigF, sigH* (20)
12	Stress survival	*ahpC, bsh, clpC, clpE, clpP, katA, msrA/BpilB, recN, sodA, sodB, ureA, ureB* (12)	*ahpC, bsh, clpC, clpE, clpP, katA, msrA/BpilB, recN, sodA, sodB, ureA, ureB* (12)

Notes: The putative virulence genes were predicted in the FJAT-10508 and FJAT-13563 genomes using the Virulence Factor Database (VFDB) (Liu et al., 2022).

Liu B, Zheng D, Zhou S, Chen L, Yang J. VFDB 2022: a general classification scheme for bacterial virulence factors. Nucleic Acids Res, 2022, 50(D1):D912-D917.

Table S3. Putative ARGs predicted in the FJAT-10508 and FJAT-13563 genomes

NO.	Functioin description	FJAT-10508	FJAT-13563
1	aminocoumarin antibiotic	*cpxR, gyrB, abeS, baeR, mdtB, novA* (6)	*gyrB, abeS, baeR, mdtB, novA* (5)
2	aminoglycoside antibiotic	*rpsL, tlyA, pmpM, cpxR, ranA, lmrS, aadK, baeR, kdpD, smeR, ykkC, ykkD* (12)	*rpsL, tlyA, pmpM, ranA, lmrS, baeR, kdpD, smeR, ykkC, ykkD* (10)
3	antibacterial free fatty acids	*mtrR* (1)	*mtrR* (1)
4	bicyclomycin-like antibiotic	*bcr-1* (1)	*bcr-1* (1)
5	carbapenem	*CAR-1, pbp1, PBP2, PEDO-1, cpxR, PBP1a, PBP2b, PBP2x, golS, mecA* (10)	*CAR-1, pbp1, PBP2, PEDO-1, PBP1a, PBP2b, PBP2x, golS, mecA* (9)
6	cephalosporin	*ADC-83, BcII, acrR, pbp1, PBP2, cpxR, SRT-1, PBP1a, PBP2b, PBP2x, golS, mecA, mgrA, smeR* (14)	*ADC-83, BcII, acrR, pbp1, PBP2, SRT-1, PBP1a, PBP2b, PBP2x, golS, mecA, mgrA,* smeR (13)
7	cephamycin	*pbp1, PBP2, cpxR, PBP1a, PBP2b, PBP2x,*	*pbp1, PBP2, PBP1a, PBP2b, PBP2x,*

		golS, mecA, smeR (9)	*golS, mecA, smeR* (8)
8	diaminopyrimidine antibiotic	*cpxR, lmrS, dfrG, rsmA* (4)	*lmrS, dfrG, rsmA* (3)
9	disinfecting agents and antiseptics	*acrR, fabG, fabI, kasA, pmpM, abeM, arlR, arlS, blt, mgrA, qacE, qacL, sdrM* (13)	*acrR, fabG, fabI, kasA, pmpM, abeM, arlR, arlS, blt, mgrA, qacE, qacL* (12)
10	elfamycin antibiotic	*EF-Tu, facT* (2)	*EF-Tu, facT* (2)
11	fluoroquinolone antibiotic	*acrR, pmpM, cpxR, gyrA, norA, parC, parE, abeM, arlR, arlS, blt, efmA, efrA, evgA, evgS, mgrA, patA, patB, pmrA, qacA, rsmA, sdrM* (22)	*acrR, pmpM, gyrA, norA, parC, parE, abeM, arlR, arlS, blt, efmA, efrA, evgA, evgS, mgrA, patA, patB, pmrA, qacA, rsmA* (20)
12	fusidane antibiotic	*fusA, fusE* (2)	*fusA, fusE* (2)
13	glycopeptide antibiotic	*murG, D-Ala-D-Ala ligase, rpld, vanH, vanH1, vanH2, vanR, vanR1, vanR2, vanR3, vanR4, vanR5, vanR6, vanS, vanS1, vanS2, vanS3, vanT, vanXY* (19)	*murG, D-Ala-D-Ala ligase, rpld, vanH, vanH1, vanH2, vanR, vanR1, vanR2, vanR3, vanR4, vanS, vanS1, vanS2, vanT, vanXY* (16)
14	glycylcycline	*acrR, adeR* (2)	*acrR, adeR* (2)
15	isoniazid-like antibiotic	*fabI, kasA, mshA, ndh* (4)	*fabI, kasA, mshA, ndh* (4)
16	lincosamide antibiotic	*ermQ, cfrA, clbA, lmrB, lmrD, lsaC, vgaC, vmlR* (8)	*ermQ, cfrA, clbA, lmrB, lmrD, lsaC, vgaC, vmlR* (8)
17	macrolide antibiotic	*ermQ, rpld, cpxR, lmrS, abeS, efmA, efrA, evgA, evgS, macB, mphI, mreA, mtrR, oleC, oleD, oleI* (16)	*ermQ, rpld, lmrS, abeS, efmA, efrA, evgA, evgS, gimA, macB, mreA, mtrR, oleC, oleI* (14)
18	monobactam	*pbp1, PBP2, cpxR, PBP1a, PBP2b, PBP2x, golS, mecA* (8)	*pbp1, PBP2, PBP1a, PBP2b, PBP2x, golS, mecA* (7)
19	mupirocin-like antibiotic	*ileS, mupA* (2)	*ileS, mupA* (2)
20	nitrofuran antibiotic	*nfsA* (1)	*nfsA* (1)
21	nitroimidazole antibiotic	*frxA, msbA* (2)	*frxA, msbA* (2)
22	nucleoside antibiotic	*SAT-4, tmrB* (2)	*SAT-4, tmrB* (2)
23	nybomycin-like antibiotic	*gyrA* (1)	*gyrA* (1)
24	oxazolidinone antibiotic	*lmrS, cfrA, clbA, mlaF, optrA* (5)	*lmrS, cfrA, clbA, mlaF, optrA* (5)
25	penam	*bcII, bla1,tetA, acrR, soxS, pbp1, PBP2, PBP1a, PBP2b, PBP2x, evgA, evgS, golS, mecA, mgrA, mtrR, smeR* (17)	*bcII, bla1, acrR, pbp1, PBP2, PBP1a, PBP2b, PBP2x, evgA, evgS, golS, mecA, mgrA, mtrR, smeR* (15)
26	penem	*soxS, golS* (2)	*golS* (1)

27	peptide antibiotic	*mprF, rpoB, yybT, liaR, liaS, liaR1, liaS1, pmrF, cpxR, cls, menA, pgsA, rpoC, walK, cdsA, yojI, bacA, bcrA, bcrB, bcrC, cprR, mgrA, rosA, rosB, ugd* (25)	*mprF, rpoB, yybT, liaR, liaS, liaR1, liaS1, pmrF, cls, menA, pgsA, rpoC, walK, cdsA, yojI, almE, bacA, bcrA, cprR, mgrA, rosA, rosB, ugd* (23)
28	phenicol antibiotic	*acrR, soxS, LmrS, cfrA, clbA, cmlv, cmx, fexA, golS, optrA, rsmA, ykkC, ykkD* (13)	*acrR, lmrS, cfrA, clbA, cmlv, cmx, fexA, golS, optrA, rsmA, ykkC, ykkD* (12)
29	phosphonic acid antibiotic	*abaF, ptsI, fosA7.5, fosM2, glpT, murA* (6)	*abaF, PtsI, fosA7.5, glpT, murA* (5)
30	pleuromutilin antibiotic	*taeA, cfrA, clbA, lsaC, vgaC, vgaD* (6)	*taeA,cfrA,clbA,lsaC,vgaC,vgaD* (6)
31	pyrazine antibiotic	*rpsA* (1)	*rpsA* (1)
32	rifamycin antibiotic	*rpoB, acrR, helR, rox, efrA, rphA, rphB* (7)	*rpoB, acrR, helR, rox, efrA, rphA, rphB* (7)
33	salicylic acid antibiotic	*thyA* (1)	*thyA* (1)
34	streptogramin A antibiotic	*ermQ, cfrA, clbA, vatF, vgaC, vgaD* (6)	*ermQ, vcfrA, clbA, vgaC, vgaD* (5)
35	streptogramin B antibiotic	*ermQ, vmlR* (2)	*ermQ, vmlR* (2)
36	streptogramin antibiotic	*ermQ, cfrA, clbA, lsaC, vatF, vgaC, vgaD, vmlR* (8)	*ermQ, cfrA, clbA, lsaC, vgaC, vgaD, vmlR* (7)
37	sulfonamide antibiotic	*cpxR, sul4* (2)	*sul4* (1)
38	tetracycline antibiotic	*tetA, acrR, cpxR, txR, adeR, evgA, evgS, mgrA, rpsJ, tet(30), tet(39), tet(L), tet(T), tetA(58), tetA(60), tetB(60), ykkC, ykkD* (18)	*acrR, txR, adeR, evgA, evgS, mgrA, rpsJ, tet(30), tet(35), tet(39), tet(L), tet(T), tetA(58), tetA(60), tetB(60), ykkC, ykkD* (17)

Notes: The putative antibiotic-resistance genes were predicted in the FJAT-10508 and FJAT-13563 genomes using the Comprehensive Antibiotic Resistance Database (CARD) (Alcock et al., 2023).

Alcock BP, Huynh W, Chalil R, Smith KW, Raphenya AR, Wlodarski MA, Edalatmand A, Petkau A, Syed SA, Tsang KK, Baker SJC, Dave M, McCarthy MC, Mukiri KM, Nasir JA, Golbon B, Imtiaz H, Jiang X, Kaur K, Kwong M, Liang ZC, Niu KC, Shan P, Yang JYJ, Gray KL, Hoad GR, Jia B, Bhando T, Carfrae LA, Farha MA, French S, Gordzevich R, Rachwalski K, Tu MM, Bordeleau E, Dooley D, Griffiths E, Zubyk HL, Brown ED, Maguire F, Beiko RG, Hsiao WWL, Brinkman FSL, Van Domselaar G, McArthur AG. CARD 2023: expanded curation, support for machine learning, and resistome prediction at the Comprehensive Antibiotic Resistance Database. Nucleic Acids Res, 2023, 51(D1):D690-D699.

Table S4. Statistics of transferable elements carrying virulence and resistance genes in the FJAT-10508 and FJAT-13563 genomes

geneID	strat	end	ARG	VF	mobile genetic elements
**FJAT-10508**					
gene0227	201799	202671	*patA*	/	Chromosome:compositional_outlier(199645:210182)
gene0229	203942	204625	*vanR* gene in *vanG* cluster	/	Chromosome:compositional_outlier(199645:210182)
gene0231	206465	207229	*macB*	Immune modulation	Chromosome:compositional_outlier(199645:210182)
gene0256	228237	229571	*glpT*	/	Chromosome:compositional_outlier(222379:230203)
gene0520	506342	507745	*abaF*	/	Chromosome:compositional_outlier(497404:525283)
gene0521	508085	508399	*mgrA*	/	Chromosome:compositional_outlier(497404:525283)
gene0798	787150	788070	*bcrA*	Exotoxin	Chromosome:compositional_outlier(787335:792969)
gene0799	788111	788800	*bcrB*	/	Chromosome:compositional_outlier(787335:792969)
gene0800	788897	789496	*bcrC*	/	Chromosome:compositional_outlier(787335:792969)
gene0801	789869	790582	*vanR* gene in *vanF* cluster	Regulation	Chromosome:compositional_outlier(787335:792969)
gene0802	790557	791597	*vanS* gene in *vanF* cluster	Regulation	Chromosome:compositional_outlier(787335:792969)
gene1195	1154893	1155807	*bla1*	/	Chromosome:compositional_outlier(1149543:1155319)
gene1232	1192121	1192792	*vanR* gene in *vanC* cluster	Regulation	Chromosome:compositional_outlier(1192026:1210101)
gene1233	1192789	1194147	*vanS* gene in *vanM* cluster	Regulation	Chromosome:compositional_outlier(1192026:1210101)
gene1234	1194267	1195202	*bcrA*	/	Chromosome:compositional_outlier(1192026:1210101)
gene1237	1196189	1197439	*liaS*	/	Chromosome:compositional_outlier(1192026:1210101)
gene1238	1197451	1198116	*liaR*	Effector delivery	Chromosome:compositional_outlier(1192026:121010

				system	1)
gene1241	1201750	1203897	*lmrD*	Exotoxin	Chromosome:compositional_outlier(1192026:1210101)
gene1593	1589206	1589946	*fabG*	Motility	Chromosome:compositional_outlier(1589781:1595377)
gene1594	1590031	1590264	/	Immune modulation	Chromosome:compositional_outlier(1589781:1595377)
gene1597	1594834	1595823	/	Motility	Chromosome:compositional_outlier(1589781:1595377)
gene1986	2092412	2092837	/	Immune modulation	Chromosome:compositional_outlier(2089732:2105287)
gene2220	2310105	2325719	/	Immune modulation	Chromosome:compositional_outlier(2312254:2317549)
gene2424	2528798	2530054	*efmA*	/	Chromosome:compositional_outlier(2529744:2542744)
gene2442	2541742	2543040	*kdpD*	/	Chromosome:compositional_outlier(2529744:2542744)
gene3546	3621818	3622753	*vanS* gene in *vanM* cluster	Regulation	Chromosome:compositional_outlier(3619716:3627813)
gene3547	3622754	3623452	*vanR* gene in *vanI* cluster	Regulation	Chromosome:compositional_outlier(3619716:3627813)
gene3550	3625300	3626244	*bcrA*	Exotoxin	Chromosome:compositional_outlier(3619716:3627813)
gene3552	3627027	3627875	/	Immune modulation	Chromosome:compositional_outlier(3619716:3627813)
gene3553	3627896	3628843	/	Immune modulation	Chromosome:compositional_outlier(3619716:3627813)
**FJAT-13563**					
gene0238	212169	212822	*liaR*	Regulation	Chromosome:compositional_outlier(209944:220076)
gene0239	213040	213972	*tetA(58)*	Exotoxin	Chromosome:compositional_outlier(209944:220076)
gene0243	216667	217362	/	Exotoxin	Chromosome:compositional_outlier(209944:220076)

gene0262	264272	265606	*glpT*	/	Chromosome:compositional_outlier(259748:265275)
gene0524	539069	540472	*abaF*	/	Chromosome:compositional_outlier(532080:550218)
gene0546	558495	559544	*clbA*	/	Chromosome:compositional_outlier(554529:565291)
gene0692	710197	711150	*tetA(58)*	Exotoxin	Chromosome:compositional_outlier(709558:717585)
gene0693	711151	712026	/	Exotoxin	Chromosome:compositional_outlier(709558:717585)
gene0694	712354	712824	/	Effector delivery system	Chromosome:compositional_outlier(709558:717585)
gene0757	773898	776987	*evgS*	Regulation	Chromosome:compositional_outlier(762293:775291)
gene0885	894934	896505	*tet(35)*	/	Chromosome:compositional_outlier(887241:895414)
gene0894	905820	906785	*patA*	/	Chromosome:insertion_sequence(906839:908123)
gene0897	908144	909589	/	Stress survival	Chromosome:insertion_sequence(906839:908123)
gene1388	1360341	1361087	*fabG*	/	Chromosome:compositional_outlier(1359603:1365087)
gene1854	1919288	1919908	*patA*	Others	Chromosome:compositional_outlier(1909715:1922516)
gene2069	2175415	2175840	/	Immune modulation	Chromosome:compositional_outlier(2174609:2192529)
gene2090	2192316	2192888	/	Motility	Chromosome:compositional_outlier(2174609:2192529)
gene2365	2439391	2455005	/	Immune modulation	Chromosome:compositional_outlier(2442292:2450093)
gene2571	2658239	2659495	*efmA*	/	Chromosome:compositional_outlier(2659509:2665023)
gene3414	3484108	3485445	*qacA*	/	Chromosome:compositional_outlier(3484937:3522596)
gene3419	3489176	3490918	*novA*	Exotoxin	Chromosome:compositional_outlier(3484937:3522596)
gene3421	3502445	3519544	*almE*	/	Chromosome:compositional_outlier(3484937:3522596)

Notes: The mobilome (plasmids, prophages, insertion sequences, inverted repeat elements, and compositional outlier regions) annotation of the FJAT-10508 and FJAT-13563 genomes was performed by using the Mobilome Annotation Pipeline developed by EBI-Metagenomics (https://github.com/

EBI-Metagenomics/mobilome-annotation-pipeline).

Table S5. Pangenome categories of the 435 *B. elezensis* genomes

Pangenome categories	Criteria	Gene count	Percentage
Strict core genes	(strains = 100%)	411	1.48%
Core genes	(99% <= strains < 100%)	1630	5.87%
Soft core genes	(95% <= strains < 99%)	954	3.43%
Shell genes	(15% <= strains < 95%)	1440	5.18%
Cloud genes	(0% <= strains < 15%)	23356	84.04%
Total genes	(0% <= strains <= 100%)	27791	100.00%

Notes: The pangenome analysis of 435 *B. velezensis* genomes (including all NCBI complete genomes and FJAT-10508 and FJAT-13563 genomes) was performed using PGAP2 (https://github.com/bucongfan/PGAP2) (Bu et al., 2025).

Bu C, Zhang H, Zhang F, Liang W, Gao H, Zhao J, Lv F, Xue R, Liu Q, Zhang Z, Jin Z, Xiao J. PGAP2: A comprehensive toolkit for prokaryotic pan-genome analysis based on fine-grained feature networks. Nat Commun. 2025, 16(1):9865.

Table S6. Cytotoxicity assays of the FJAT-10508 and FJAT-13563 culture supernatants to the Caco-2 cells

Treatment	The viability rate of cells
PBS+cell	99.72 ± 0.91%
TritonX-100+cell	5.8% ± 0.5%
FJAT-10508 supernatant+cell	198.71 ± 3.56 %
FJAT-13563 supernatant+cell	185.56 ± 4.05 %

Note: Cell viability was calculated using PBS + cell as the negative control. Triton X-100 (1%) served as a positive control for complete cell lysis.
